# *PpePL1* and *PpePL15* Are the Core Members of the Pectate Lyase Gene Family Involved in Peach Fruit Ripening and Softening

**DOI:** 10.3389/fpls.2022.844055

**Published:** 2022-03-25

**Authors:** Ze Xu, Jieyu Dai, Tongyang Kang, Kamran Shah, Qin Li, Kun Liu, Libo Xing, Juanjuan Ma, Dong Zhang, Caiping Zhao

**Affiliations:** College of Horticulture, Northwest Agriculture and Forestry University, Yangling, China

**Keywords:** peach (*Prunus persica* (L.) Batsch), softening, pectate lyase, VIGS, fruit

## Abstract

Pectin is the major component in the primary cell wall and middle lamella, maintaining the physical stability and mechanical strength of the cell wall. Pectate lyase (PL), a cell wall modification enzyme, has a major influence on the structure of pectin. However, little information and no comprehensive analysis is available on the PL gene family in peach (*Prunus persica* L. Batsch). In this study, 20 *PpePL* genes were identified in peach. We characterized their physicochemical characteristics, sequence alignments, chromosomal locations, and gene structures. The PpePL family members were classified into five groups based on their phylogenetic relationships. Among those, *PpePL1, 9, 10, 15,* and *18* had the higher expression abundance in ripe fruit, and *PpePL1, 15,* and *18* were upregulated during storage. Detailed RT-qPCR analysis revealed that *PpePL1* and *PpePL15* were responsive to ETH treatment (1 g L^−1^ ethephon) with an abundant transcript accumulation, which suggested these genes were involved in peach ripening and softening. In addition, virus-induced gene silencing (VIGS) technology was used to identify the roles of *PpePL1* and *PpePL15*. Compared to controls, the RNAi fruit maintained greater firmness in the early storage stage, increased acid-soluble pectin (ASP), and reduced water-soluble pectin (WSP). Moreover, transmission electron microscopy (TEM) showed that cell wall degradation was reduced in the fruit of RNAi-1 and RNAi-15, which indicated that softening of the RNAi fruit has been delayed. Our results indicated that *PpePL1* and *PpePL15* play an important role in peach softening by depolymerizing pectin and degrading cell wall.

## Introduction

Peach (*Prunus persica* L. Batsch), a typical climacteric fruit, is one of the most important economic fruit crops in temperate regions (Yoshioka et al., 2010). However, peach fruit undergoes rapid softening after postharvest, which limits the shelf-life and decreases market values. Therefore, studying the molecular mechanisms of softening in peach will benefit the commercialization of the fruit.

Fruit softening is mainly caused by modification or remodeling of the cell wall (Brummell et al., 2004; Wang et al., 2018). Pectin is the major component in the primary cell wall and middle lamella, maintaining the physical stability and mechanical strength of the cell wall, consisting of homogalacturonan (HG), rhamnogalacturonan I (RG-I), the substituted galacturonans rhamnogalacturonan II (RG-II), and xylogalacturonan (XGA; Ridley et al., 2001). Fruit softening is accompanied by the depolymerization of pectins, solubilization of pectin polymers, and loss of neutral sugars from pectin side chains (Ruiz-May and Rose, 2013; Tucker, 2014). Many studies have reported that cell wall-modifying enzymes play a vital role in depolymerization and solubilization of pectin, including polygalacturonase (PG, EC3.2.1.15), β-galactosidase (β-gal, EC3.2.1.23), pectin methylesterases (PME, EC3.1.1.11), xyloglucan endotransglucosylase/hydrolase (XTH, EC:2.4.1.207), and pectate lyase (PL, EC4.2.2.2; Brummell et al., 2004; Qian et al., 2016; Uluisik et al., 2016; Zhang et al., 2017; Liu et al., 2018). These enzymes acted synergistically to degrade pectin. When the methylated HG was secreted to the apoplast, PMEs removed the methoxyl residues from the methylated HG to produce HG, which can be hydrolyzed by PG and PL (Wang et al., 2018).

Pectate lyase (PL) belongs to polysaccharide lyase family 1 (PL1) and cleavage of polymers of a-1,4 galactosyluronic acid molecules of pectins from the middle lamella and primary cell walls (Marin-Rodriguez et al., 2002). PL family members have been identified in *Arabidopsis thaliana*, rice, cotton, *Brassica rapa*, poplar, and tomato (Palusa et al., 2007; Sun and Nocker, 2010; Cao, 2012; Jiang et al., 2013; Bai et al., 2017; Yang et al., 2017; Sun et al., 2018; Zheng et al., 2018). Since PL was first reported in *Erwinia carotovora* and *Bacillus* sp. (Starr and Moran, 1962), a large number of researches have shown that PL has played a role in multiple biological processes of plant growth and development, including pollen development (Jiang et al., 2014; Zheng et al., 2018), fruit ripening and softening (Marin-Rodriguez et al., 2002; Payasi and Sanwal, 2003; Uluisik et al., 2016; Yang et al., 2017), petal abscission (Singh et al., 2011), leaf development and senescence (Leng et al., 2017), fiber elongation (Wang et al., 2010), vascular tissues development (Bai et al., 2017), and disease resistance (Vogel et al., 2002).

The role of PL in manipulating fruit softening has been reported in climacteric fruit (banana, tomato, mango) and non-climacteric fruit (strawberry and grape; Nunan et al., 2001; Pua et al., 2001; Jimenez-Bermudez et al., 2002; Chourasia et al., 2006; Uluisik et al., 2016). PL activity increased gradually during banana fruit ripening and peaked at a climacteric peak, accompanied by an increase in soluble polyuronides (Payasi and Sanwal, 2003; Payasi et al., 2006). Moreover, pectate lyase transcripts were accumulated in ripe fruit and were affected by exogenous ethylene (Pua et al., 2001; Chourasia et al., 2006). In strawberry, *PL* genes were also mainly expressed in ripe fruit (Benitez-Burraco et al., 2003). These results suggest a close relationship between fruit ripening and *PL* genes.

Furthermore, antisense inhibition of *PL* gene expression in strawberry delayed fruit softening, pectin solubility was reduced, and depolymerization of tightly bound polyuronides was decreased (Santiago-Domenech et al., 2008). In tomato, silencing *PL* inhibited fruit softening, prolonged shelf-life, reduced the content of water-soluble pectins (WSP), and increased pathogen resistance (Uluisik et al., 2016; Yang et al., 2017). These researches demonstrated that PL plays an important role in fruit softening by degrading pectic polysaccharides. In peach, PL activity was found to have an inverse relationship with fruit firmness during storage (Ortiz and Lara, 2008). However, the exact function of PL family members in peach softening is still unclear.

In this study, we identified PL family members associated with peach fruit softening. Virus-induced gene silencing technology (VIGS) was used to clarify the function of *PpePL1* and *PpePL15* in peach fruit softening. Our research provides a foundation for further investigation of the function of PL family members in peach fruit ripening and softening.

## Materials and Methods

### Plant Materials and Treatment

Peach (*Prunus persica* L. Batsch) cv. “Qian Jian Bai” (QJB) and “Zao Feng Wang” (ZFW) were planted in the Experimental Station of the College of Horticulture at Northwest A & F University, Yangling, Shaanxi, China. In the commercial harvest period, about 100 “ZFW” fruit with the same maturity, high hardness, uniform size, and no mechanical damage was random harvested and stored in a storage room with a temperature of 25 ± 1°C and a relative humidity of 75–85%. The “ZFW” fruit was divided into three subgroups for three biological replications. Peach flesh samples were collected every 2 days until the fruit was completely softened. After 8 days (times after harvest), the “ZFW” fruit was completely softened and was collected at 5 time points (0, 2, 4, 6, and 8 days). Samples (mixing flesh samples from five fruit) were frozen with liquid nitrogen and stored at −80°C.

For postharvest treatment, about 300 “QJB” fruits were collected and evenly divided into three groups. Following the method described by Qian et al. (2016), three treatments were carried out, including ETH treatment (1 g L^−1^ ethephon, 15 min), 1-MCP treatment (5 μl L^−1^1-Methylcyclopropene, 24 h), and CK (control, water, 15 min). All treated fruit were stored in a storage room with a temperature of 25 ± 1°C. The “QJB” fruits were stored for 6 days and were collected at 4 time points (0, 2, 4, and 6 days). Each treatment was divided into three subgroups for three biological replications. Samples (mixing flesh samples from five fruit) were frozen with liquid nitrogen and stored at −80°C.

### Identification and Analysis of PpePL Family Members

To identify PpePL family members, first, we used 26 AtPL protein sequences from *Arabidopsis* genome (the AtPL protein sequence were listed in Supplementary Table S3) as queries to search the peach genome database (*Prunus persica* Genome v2.0.a1)^1^; then, the Hidden Markov Model (HMM) profile of the Pec_lyase_C domain (Pfam00544) was retrieved from the Pfam database (Finn et al., 2008),^2^ and the E-value threshold for the HMMER (version 3.2.1) was set at 1 × e^−10^ to obtain possible PL proteins (Sun et al., 2018). All candidate PL family members were manually checked using CD-search^3^ to confirm the presence of the PL conserved domain. The online website ExPASY^4^ was used to predict protein physicochemical characteristics including protein length, molecular weight, isoelectric points, instability index, aliphatic index, and grand average of hydropathicity (GRAVY; Wilkins et al., 1999). Signal peptides were analyzed by Signal 4.0 (Petersen et al., 2011). The PpePL chromosome locations were obtained from the genome sequence database, and then genes were mapped to the chromosome using MapDraw (Liu and Meng, 2003).

### Molecular Cloning of PpePL Family Members

To clone the coding sequence (CDS) of PpePL members, the cDNAs from fruit of cv. “ZFW” were used as templates for PCR amplification. Specific primers were designed based on published genome sequences by Primer Premier 6.0 software. Primer sequences are listed in Supplementary Table S1. The full-length CDS of *PpePL* genes was amplified using Phanta HS Super-Fidelity DNA Polymerase (Vazyme, Nanjing, China). The PCR products were inserted into the pMD18-T vector (TaKaRa, Dalian, China) and transferred into competent *Escherichia coli* cells (Tiangen Biotech, Beijing, China). Then, the positive clones were sequenced with M13-F and M13-R. The cloned sequences were listed in Supplementary Figure S1.

### Sequence Alignment, Phylogenetic Analysis, Gene Structure, and Motif Identification

The full-length deduced amino acid sequences of PpePL family members were used for multiple alignment by DNAMAN (version 6.0). The PL protein sequence of tomato, *Arabidopsis*, and other plants was obtained through the tomato genome,^5^ TAIR,^6^ and NCBI,^7^ respectively. A phylogenetic tree was constructed using MEGA 6.0 with a neighbor-joining (NJ) method. The Bootstrap test was set at 1,000 to assess the reliability of the tree (Tamura et al., 2013). The tree was drawn using the EvolView tool (He et al., 2016). The exon and intron structures of PpePLs were analyzed by the Gene Structure Display Server 2.0 software (Hu et al., 2014).^8^ The conserved motifs of the PpePL proteins were identified using the MEME platform (Bailey et al., 2006). TBtools were used to draw the image (Chen et al., 2020). All protein sequences with gene IDs in the phylogenetic tree are listed in Supplementary Table S3.

### RNA Extraction and Expression Analysis of PpePL Family Members

Total RNA was extracted using the RNAprep Pure Plant Kit (Polysaccharides & Polyphenolics-rich; Tiangen Biotech, Beijing, China) according to the manufacturer’s protocol. About 0.3 g frozen “ZFW” and “QJB” fruit flesh were used for RNA extraction. RNA integrity and quality were tested by electrophoresis in 1% agarose gels and an ultraviolet spectrophotometer (Thermo NanoDrop 2000, Wilmington, DE, United States), respectively. Also, first-strand cDNA was synthesized *via* reverse transcription of 1 μg of total RNA using the PrimeScript RT Reagent Kit with gDNA Eraser (Takara, Dalian, China). The expression levels of *PpePLs* in “QJB” fruit during storage were obtained from previously reported RNA-seq data (Qian et al., 2016). The FPKM values of *PpePL* genes in RNA-seq data were listed in Supplementary Table S2. Expression profiles were displayed by heatmap and were drawn by TBtools (Chen et al., 2020). The primers for real-time quantitative PCR (RT-qPCR) were designed using Primer Premier 6.0 (Supplementary Table S1). RT-qPCR was carried out with the Bio-Rad CFX system (Bio-Rad, CA, United States) using a SYBR Green-based PCR assay. The 10 μl reaction volume was used for each sample, containing 2 μl ddH_2_O, 1 μl of each primer (5 μM), 1 μl cDNA (10 ng/μl) and 5 μl of SYBR Premix ExTaq II (TaKaRa, Dalian, China). The PCR amplification procedure was set as follows: 1 min at 95°C, followed by 40 cycles of 15 s at 95°C, 20 s at 60°C (annealing temperature) and 20 s at 72°C, followed by 10 s at 95°C, followed by 40 cycles to construct a melting curve. Each RT-qPCR analysis was carried out in triplicate. A no-template control (NTC) was also included in each run for each gene. *PpCYP2* (Prupe.8G233900) and *PpTua5* (Prupe.6G004100) were used as reference genes (Tong et al., 2009; Xu et al., 2022). The relative expression level was calculated by the 2^−ΔΔ*C*t^ method (Livak and Schmittgen, 2001). Melting curve of 5 *PpePL* genes and reference genes by RT-qPCR was shown in Supplementary Figure S2.

### Virus-Induced Gene Silencing

Virus-induced gene silencing technology was used to downregulate the expression of *PpePL1* and *PpePL15* in fruit. The specific cloning primers were designed by Primer Premier 6.0 software (Supplementary Table S1). An approximately 400 bp fragment from the un-conserved region of *PpePL1* (Prupe.1G060900) and *PpePL15* (Prupe.5G161300) was amplified using Phanta HS Super-Fidelity DNA Polymerase (Vazyme, Nanjing, China) and inserted into the pTRV2 vector. The recombinant plasmids were named pTRV2-*PpePL1* and pTRV2-*PpePL15*, respectively. The sequences of *PpePL1* and *PpePL15* used to construct vector were shown in Supplementary Figure S3. The empty plasmid pTRV1 and pTRV2 as well as the recombinant plasmid pTRV2-*PpePL1* and pTRV2-*PpePL15* were transformed into Agrobacterium strain GV3101 and incubated at 28°C until OD600 of 0.8. The *Agrobacterium tumefaciens* was resuspended using MES buffer (containing 10 mM MES, 10 mM MgCl_2_, and 150 mM acetosyringone) to an OD_600_ of 0.8 and stored at room temperature for 2 h as described by Bai et al. (2015). Agrobacterium infection was carried out at the end of the second exponential growth stage according to the protocols from Li et al. (2017). When fruit is half-ripe, the injection was done in the middle of the peach fruit. A total of 600 intact “ZFW” fruit with the same maturity, high hardness was selected randomly for injection. Among them, 200 fruit were injected with pTRV1 + pTRV2- *PpePL1* for *PpePL1* silencing (RNAi-1), 200 fruit were injected with pTRV1 + pTRV2- *PpePL15* for *PpePL15* silencing (RNAi-15), and 200 fruit were injected with pTRV1 + pTRV2 as the control. One week after the injection, the injected peach fruit was harvested and stored. During storage, the infected “ZFW” fruit was completely softened after 8 days (times after harvest), and peach flesh samples were collected at 5 time points (0, 2, 4, 6, and 8 days). Fruit firmness, texture parameters, enzyme activity, and gene expression were analyzed by the infected sides. Three biological repetitions were designed, and each biological repetition was carried out by mixing flesh samples from five fruit.

### Measurement of Fruit Firmness and Texture Parameters

Fruit firmness was measured by a GY-4 penetrometer equipped with a 7.9 mm cylindrical probe (Top Instrument Co., Ltd., Hangzhou, China) after the skin was removed. Texture parameters were determined using a TA.XT *plus* texture analyzer with a P/2 crosshead (Stable Micro Systems Ltd., English), following the method described by Rahman and Al-Farsi (2005) with a minor change. Peach fruit was placed on the platform of the texture profile analysis (TPA) instrument, and the fruit arc was perpendicular to the probe. The crosshead was allowed to descend at a rate of 1 mm s^−1^ for a total deformation of 5 mm. When the compression stroke was completed, a second compression cycle was performed on the same sample. All operations were automatically controlled by the texture analyzer. And the force-time curve was automatically reported by the instrument. Six texture parameters (adhesiveness, springiness, cohesiveness, gumminess, chewiness, resilience) were obtained (Singh et al., 2013).

### Transmission Electron Microscope

Sections (1 × 2 × 3 mm^3^) were obtained from the fruit infection sites and then soaked in 2 ml of 4% (*v*/*v*) glutaraldehyde solution (pH 6.8) for 12 h. The samples were carried out as follows: washed with phosphate buffer (0.1 M, pH 6.8) for four times, subjected to an ethanol gradient elution (30, 50, 70, 80, and 90%, *v*/*v*), embedded in SPI812 resin, ultrasonically sliced with an ultrathin slicer (Leica, EM UC7, Germany), and then stained with uranyl acetate and lead citrate. The cell wall was observed using a transmission electron microscopy (TEM; TECNAI G2 SPIRIT BIO, FEI, America) using 80 KV of accelerating voltage (Ding et al., 2019).

### The Measurement of Pectin Content and PL Activity

The water-soluble pectin and acid-soluble pectin (ASP) were measured as described by He et al. (2018). The pectin content was expressed as grams of D- (+) -galacturonic acid (GalA) equivalents per kilogram of fresh weight (FW) peach flesh. The PL enzyme activity was measured using a method reported by Marin-Rodriguez et al. (2003). One unit of PL enzyme activity was defined as the production of 1 nmol unsaturated digalacturonan per minute per kg of FW peach flesh.

### Statistical Analysis

Statistical differences were determined using ANOVA, and the least significant difference (LSD) at *p* < 0.05 by SPSS 22.0. The figures were prepared using Origin 2018.

## Results

### Identification of PL Family Members in Peach

**Figure 1:**
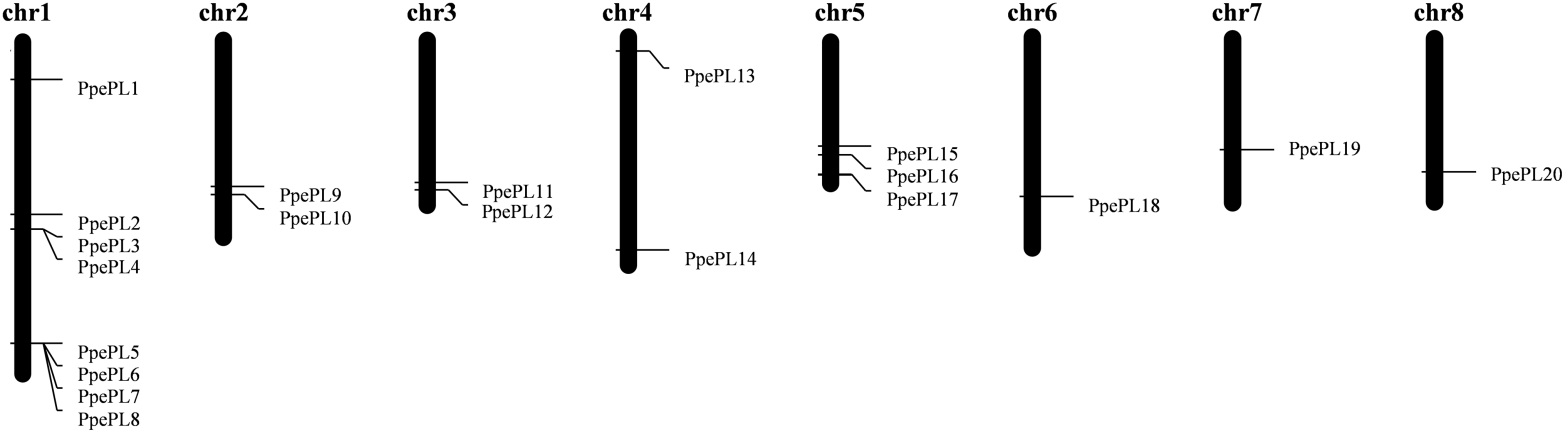
Chromosomal mapping of *PpePL* genes. chr: chromosomal.

A total of 20 genes were identified as putative PL family members (Table 1), and all of the candidates contained the PL conserved domain. According to their corresponding location on each chromosome (from top to bottom), these 20 PL members were named from *PpePL1* to *PpePL20* (Figure 1). PpePL family members were unevenly distributed among the eight chromosomes of the peach genome. Chromosomes 1 contained eight PpePL members, while chromosomes 6, 7, and 8 had only one member, respectively. There were only two PpePL members on chromosomes 2, 3, and 4, respectively, while three PpePL members were distributed on chromosomes 5. Two tandem duplication sites were found on chromosome 1.

**Table 1:** PpePL gene family with their molecular details.

Gene name	Genome v2.0	Length (aa)	MW (kDa)	PI	Signal peptide	Instability index	Aliphatic index	GRAVY
*PpePL1*	Prupe.1G060900	445	48.71	6.8	+	38.99	72.79	−0.29
*PpePL2*	Prupe.1G239900	401	43.94	8.58	+	35.93	77.83	−0.315
*PpePL3*	Prupe.1G268500	443	49.64	8.58	+	24.33	74.63	−0.309
*PpePL4*	Prupe.1G268700	446	49.89	8.95	+	29.66	72.8	−0.367
*PpePL5*	Prupe.1G565800	395	44.33	9.55	+	43.59	79.72	−0.329
*PpePL6*	Prupe.1G565700	395	44.27	9.55	+	36.95	80.46	−0.346
*PpePL7*	Prupe.1G565500	395	44.24	9.5	+	38.52	81.44	−0.336
*PpePL8*	Prupe.1G565000	382	43.25	9.46	+	35.6	78.32	−0.41
*PpePL9*	Prupe.2G206100	563	61.19	6.38	−	33.66	84.19	−0.166
*PpePL10*	Prupe.2G226100	333	37.52	6.88	−	37.6	76.76	−0.367
*PpePL11*	Prupe.3G271600	452	50.31	9.09	+	30.1	80.24	−0.269
*PpePL12*	Prupe.3G296600	405	44.55	6.84	+	41.45	77.33	−0.314
*PpePL13*	Prupe.4G017400	385	42.29	9.33	+	35.4	72.18	−0.168
*PpePL14*	Prupe.4G020600	328	36.34	8.87	+	37.47	74.94	−0.139
*PpePL15*	Prupe.5G161300	413	45.25	6.62	+	38.2	76.27	−0.32
*PpePL16*	Prupe.5G186900	440	49.25	8.85	−	28.98	78.73	−0.371
*PpePL17*	Prupe.5G245600	244	27.44	9.82	−	27.95	80.7	−0.363
*PpePL18*	Prupe.6G247100	375	41.47	8.85	−	34.03	73.6	−0.481
*PpePL19*	Prupe.7G126700	487	53.58	6	+	34.68	78.05	−0.27
*PpePL20*	Prupe.8G206800	421	46.65	7.04	+	31.54	80.64	−0.252

PpePL protein characteristics were analyzed and listed in Table 1. The length of deduced polypeptide sequences for PpePL proteins ranged from 244 to 563 amino acid residues, with predicted molecular weights between 27.44 and 61.19 kDa. The predicted isoelectric points of PpePL proteins ranged from 6 to 9.82, and the value of the aliphatic index ranged from 72.18 to 84.19. Most of the PpePL deduced proteins were stable, with instability index values less than 40 (except for PpePL5 and PpePL12). The GRAVY values of all PpePLs were less than zero, which showed that all PpePL proteins were hydrophilic. In addition, PpePL9, 10, 16, 17, and 18 did not contain signal peptide, while other members contained a signal peptide.

**Figure 2:**
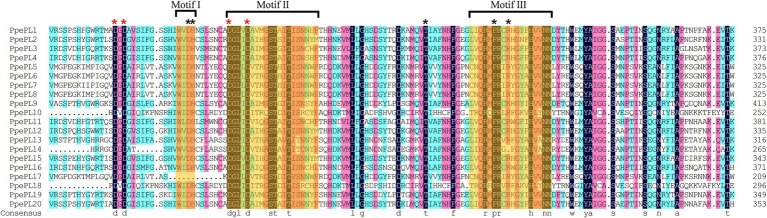
Multiple sequence alignment of Pec_lyase_C domain of PpePL protein. Orange shading indicates three typical conserved motifs of PLs, referred to as motif I, II and III. Different color indicates different similarities (black: 100%, magenta: 75%, blue: 50%). The red asterisk represents the Ca^2+^ coordination binding site, and black asterisk represents the active site residues.

Multiple sequence alignment revealed that the majority of PpePL family members contained the conserved motif I (WIDH), II (DGLIDAIMASTAITISNNYF), and III (LIQRMPRC RHGYFHVVNNDY), with the exception of PpePL17, which lacked motif I (Figure 2). The four aspartic amino acid residues corresponding to the Ca2^+^ coordination were conserved in the PpePLs protein. Five additional amino acid residues (Asp., His, Thr, Pro, and Arg) involved in catalysis and substrate binding were found to be highly conserved. Further confirming the identity of the PL family members in peach, Pfam analysis revealed that all PpePLs proteins possess a Pec_lyase_C domain. A Pec_lyase_N domain was also identified in PpePL3, 4, 11, and 16, whose structure or function has not been described.

### Phylogenetic Analysis and Gene Structure of PpePLs

**Figure 3:**
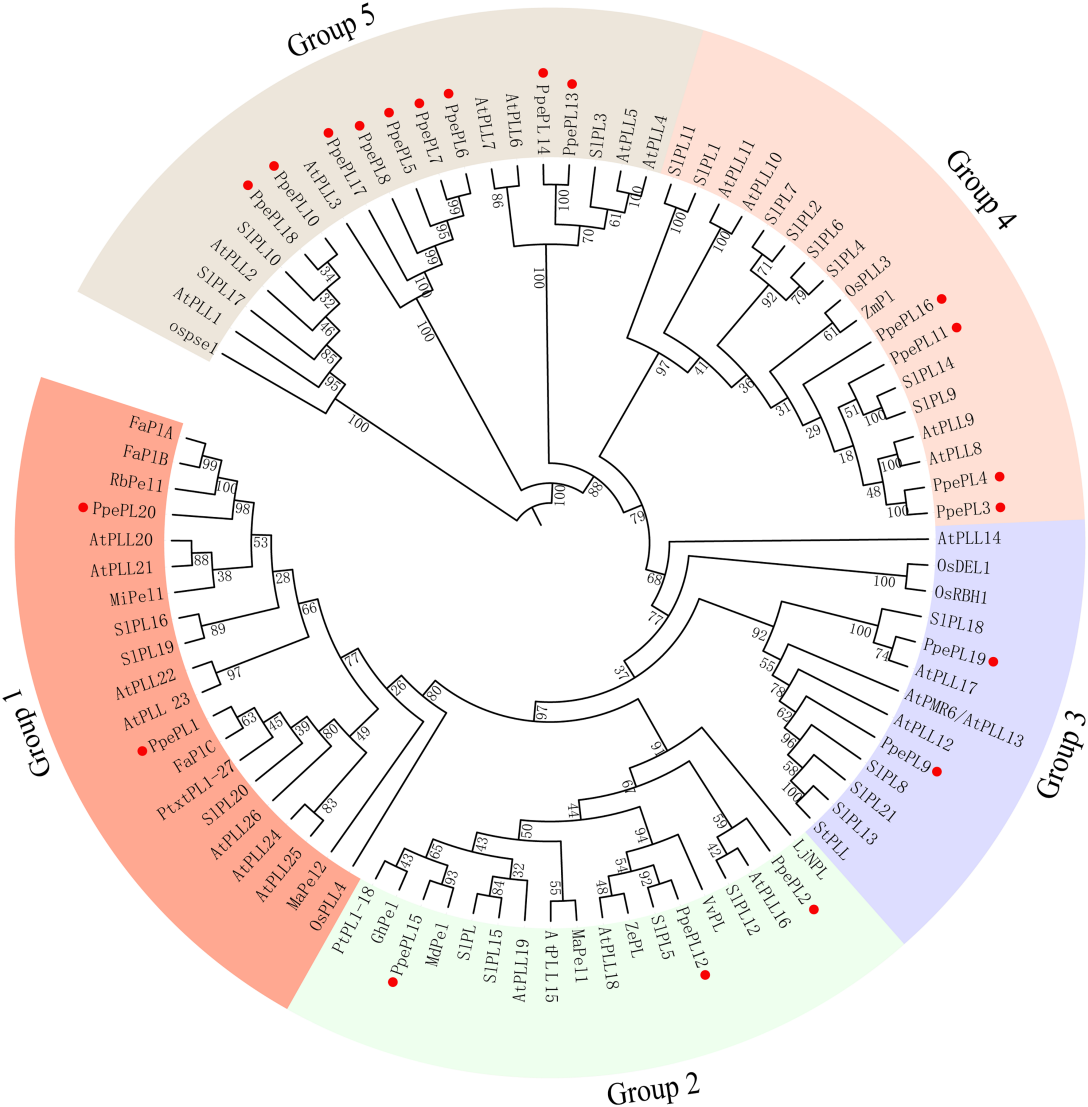
Phylogenetic tree of PLs from peach and other species. The PL proteins are clustered into five groups, marked by different colors. The red circle represents the PpePL members. Species shown are *Arabidopsis thaliana* (At), *Prunus persica* (Ppe), *Fragaria* × *ananassa* (Fa), *Musa acuminate* (Ma), *Malus domestica* (Md), *Vitis vinifera* (Vv), *Mangifera indica* (Mi), *Zinnia elegans* (Ze), *Zea mays* (Zm), *Solanum tuberosum* (St), *Rosa bourboniana* (Rb), *Lotus japonicus* (Lj), and *Solanum lycopersicun* (Sl). All protein sequences with gene IDs in the phylogenetic tree are listed in Supplementary Table S3.

According to the phylogenetic tree, the 20 PpePL genes were clustered into five groups (Figure 3). Group 5 was the largest, containing nine members of the PpePL family, while the groups 1 and 3 only consisted of two members, respectively. In group 1, PpePL1 had high homology with FaPlC, while PpePL20 had high homology with FaPlA and FaPlB. PpePL2, 12, and 15 were clustered into group 2, in which PpePL15 showed a closer relationship with MdPel and SIPL. In group 3, PpePL19 was similar to AtPLL17. PpePL3, 4, 11, and 16 with the Pec_lyase_N domain were clustered in group 4.

**Figure 4:**
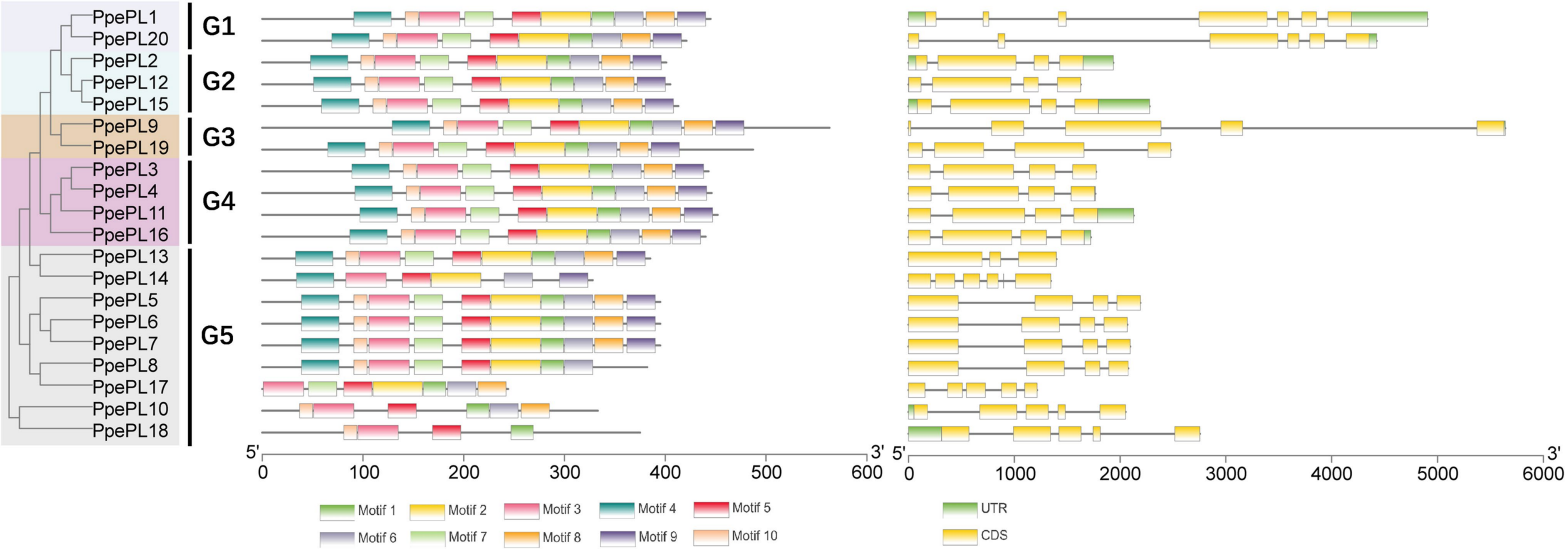
Analysis of *PpePL* gene structures. The left part indicates the unroot phylogenetic tree of PpePL proteins. Different colors represent different groups. The middle part represents conserved motif analysis by MEME website. Up to 10 motifs were shown by different colors. The right part shown the exon–intron organization of *PpePL* genes. Yellow box and black line were exon and intron positions, respectively. Green box represents the 3′-UTR in purple and 5′-UTR. CDS, coding sequence; UTR, untranslational region.

The conserved motifs of PpePL family members were identified using the online tool MEME. Most PpePL members contained 10 motifs, PpePL8 lacked motif 8 and 9, PpePL10 lacked motif 2, 4, 7 and 9, PpePL14 lacked motif 1, 7, 8 and 10, PpePL17 lacked motif 4, 9 and 10, PpePL18 lacked motif 2, 4, 6–9 (Figure 4). The results of intron/exon structures analysis showed 12 of the PpePL members contained 4 exons, and five of the PpePL members had 5 exons. While PpePL1, PpePL20, and PpePL13 contain 7, 6, and 3 exons, respectively.

### Identification of PpePL Members Related to Fruit Ripening and Softening

**Figure 5:**
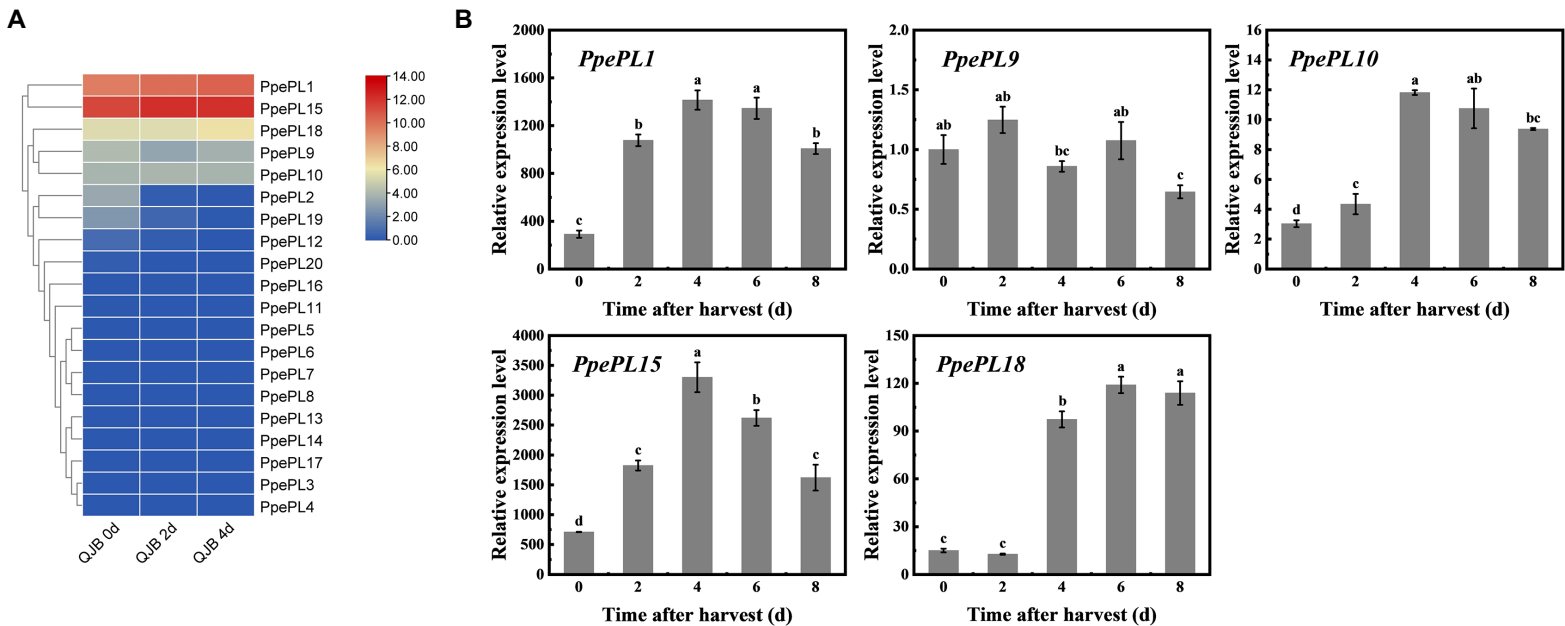
Expression profiles of the *PpePL* genes in two peach cultivars during storage. **(A)** *PpePL* gene expression level in transcriptome data of “QJB” fruit during ripening. Color scale on the right represented log_2_-transformed FPKM values. Red indicated highly expressed genes, while blue indicated low expressed genes. d indicated time after postharvest. **(B)** Relative expression of *PpePL* genes in cv. “ZFW” during ripening. *PpTua5* and *PpCYP2* served as reference gene. Each values represents the means ± standard error of three replicates. Significant differences (*p* < 0.05) between means are indicated by different letters. “QJB”: “Qian Jian Bai”; “ZFW”: “Zao Feng Wang.”

To characterize the putative function of *PpePL* members, we identified the *PpePL* genes expression level by transcriptome of “QJB” fruit during storage (Qian et al., 2016; Figure 5A). The result showed that among 20 *PpePL* genes, seven *PpePL* members were expressed in ripe fruit (*PpePL1, 2, 9, 10, 15, 18*, and *19*). Furthermore, the expression level of *PpePL2* and *PpePL19* exhibited declining trends during storage. *PpePL1, 9, 10, 15,* and *18* had the higher expression abundance in ripe fruit, among them, *PpePL1, 15,* and *18* were upregulated during storage, and *PpePL10* was relatively stable (Supplementary Table S2).

Expression levels of 20 *PpePL* members were further confirmed during “ZFW” fruit softening. Similar to the transcriptome data, only five *PpePL* genes were detectable (*PpePL1, 9, 10, 15,* and *18*), and the expression level of *PpePL2* and *PpePL19* was very low or almost undetectable. The expression of *PpePL1, 10, 15,* and *18* showed upregulation expression during storage, and *PpePL1*, *10*, *15,* reached expression peak at 4 days (time after harvest), while *PpePL18* at 6 days. *PpePL9* showed a downward expression trend (Figure 5B). Moreover, *PpePL1* and *15* exhibited the highest transcription abundance during fruit ripening and softening in two cultivars (“QJB” and “ZFW”).

**Figure 6:**
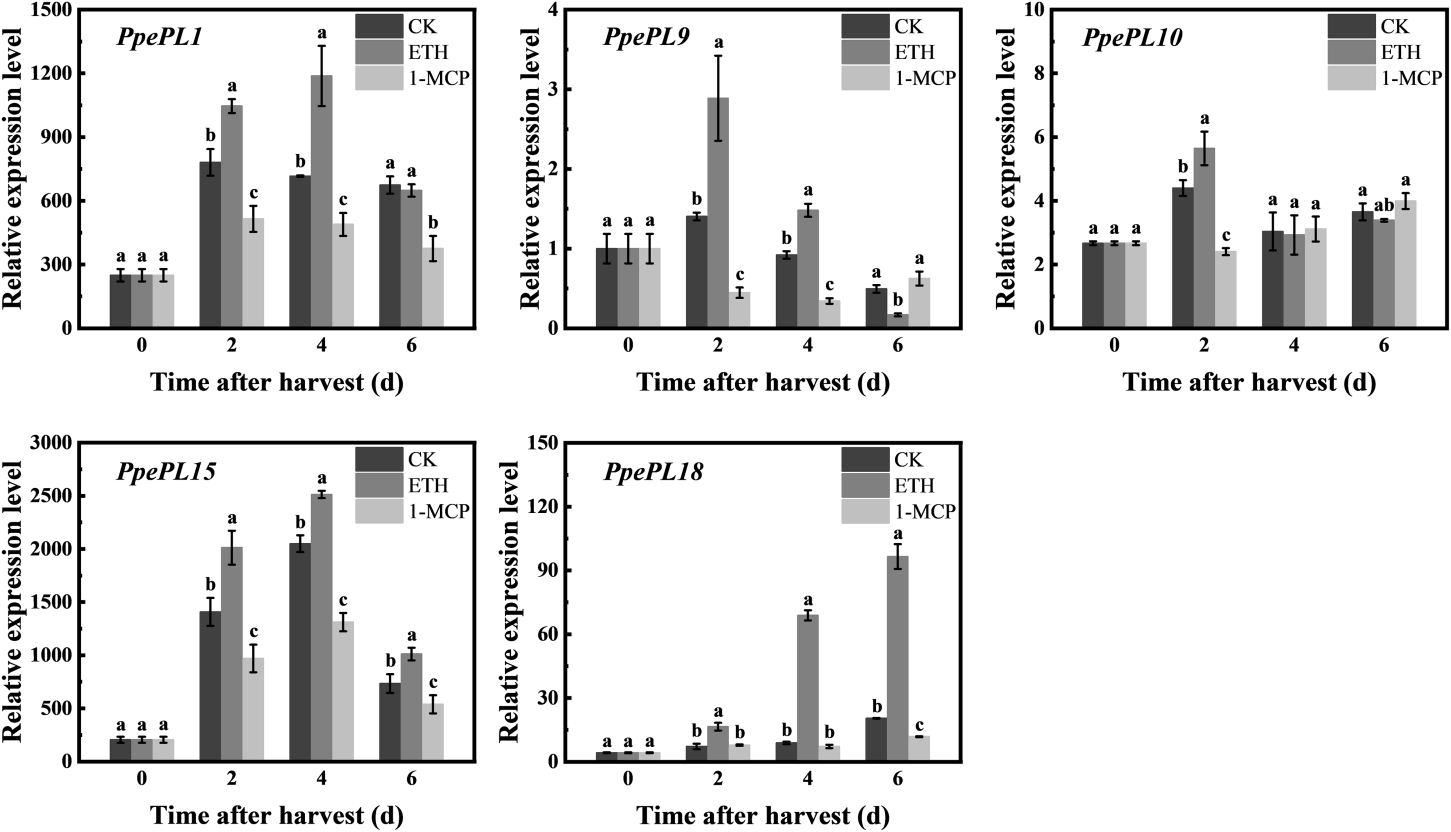
Relative expression of *PpePL1, 9, 10 15,* and *18* in response to ethylene and 1-MCP treatment in cv. “QJB” fruit during storage. *PpTua5* and *PpCYP2* served as reference gene. Each values represents the means ± standard error of three replicates. Significant differences (*p* < 0.05) between means are indicated by different letters. “QJB”, “Qian Jian Bai”; ETH, ethephon treatment; 1-MCP, 1-Methylcyclopropene treatment; CK, Control.

We also detected the response of these *PpePL* members to ethylene treatment in “QJB” fruit (Figure 6). In CK (control) fruit, *PpePL1, 15, 18* showed upregulation expression, and the peak was reached at 2, 4, and 6 days, respectively. *PpePL9* also showed a downward expression trend and *PpePL10* was relatively stable during storage (Figure 6). After ETH (ethephon) treatment, the expression levels of *PpePL1, 9, 15, 18* were upregulated at 2–4 days or 2–6 days, while *PpePL10* was only upregulated at 2 days, and their peak expression was higher than that of CK fruit. After 1-MCP (1-methylcyclopropene) treatment, expression of *PpePL1* and *15* was inhibited during storage, *PpePL9* was downregulated at 2 and 4 days, *PpePL10* was only downregulated at 2 days. *PpePL1* and *PpePL15* were likely to play an important role in peach fruit ripening and softening based on their high expression level, ripening-related expression pattern, and response to ethylene regulator treatment.

To confirm the accuracy of the putative sequences, the full-length coding sequences of *PpePL1*, *PpePL9*, *PpePL10*, *PpePL15,* and *PpePL18* were cloned by PCR amplification. Compared with the published peach gene predictions, we detected some incompatible bases in *PpePL1*, *PpePL9,* and *PpePL10* (Supplementary Figure S1). The CDS of *PpePL15* and *PpePL18* showed full identity with the corresponding genome sequence. It suggested that the cloned sequence has high similarity to the predicted CDS in the peach genome (over 99% sequence similarity).

### Downregulated *PpePL1* and *PpePL15* Affect PL Enzyme Activity, Firmness, and Texture Parameters

**Figure 7:**
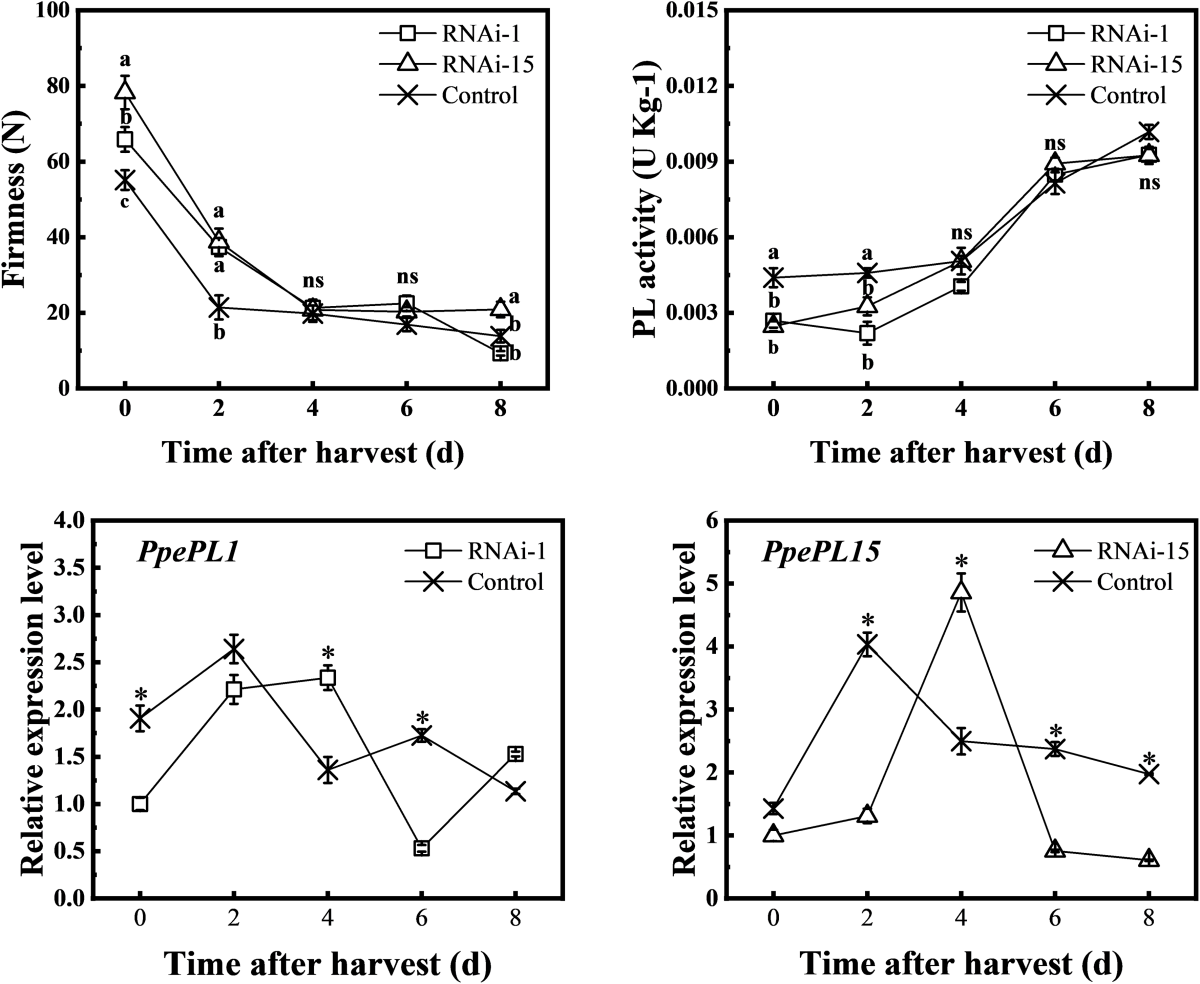
Changes in the firmness, PL activity, and relative expression of *PpePL1* and *PpePL15* during storage of control (TRV2), RNAi-1 (TRV-*PpePL1*), and RNAi-15 (TRV-*PpePL15*). Expression levels determined by RT-qPCR are relative to the expression in control (TRV2) fruit at the start of the storage period. RT-qPCR data were normalized by *PpTua5* and *PpCYP2*. Each values represents the means ± standard error of three replicates. The asterisk indicated a significant difference (*p* < 0.05), using the two-tailed Student’s *t*-test. Significant differences (*p* < 0.05) between means are indicated by different letters (**a**, **b**, and **c**).

To further confirm the function of *PpePL1* and *PpePL15* in peach softening, virus-induced gene silencing technology was used to downregulate *PpePL1* and *PpePL15* expression levels in fruit, respectively. Fruit injected with pTRV1 + pTRV2-*PpePL1/15* was a gene silencing (RNAi) fruit, and fruit injected with pTRV1 + pTRV2 was a control. Compared to control, in RNAi-1 fruit, *PpPL1* expression was suppressed at 0, 2, and 6 days. However, at 4 days *PpPL1* expression did not decrease (Figure 7). In RNAi-15 fruit, *PpPL15* expression was downregulated at 2, 6, and 8 days and was enhanced at 4 days. At the same time, PL enzyme activity in RNAi-1 and RNAi-15 fruit was lower than in control fruit in the first 2 days. In the first 2 days, the firmness of RNAi-1 and RNAi-15 fruit was higher than that of the control fruit.

**Figure 8:**
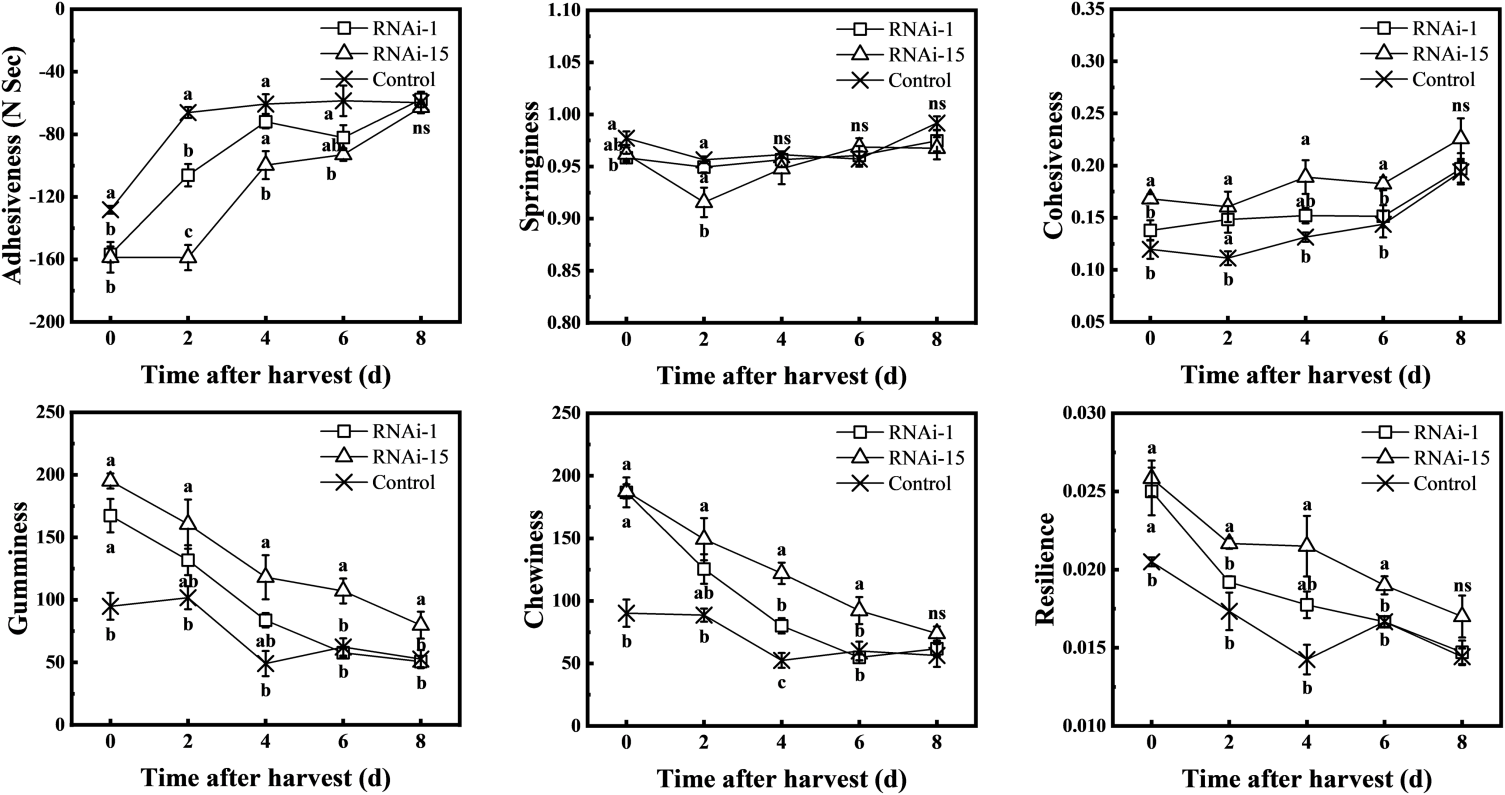
Changes in the texture profile analysis (TPA) parameters during storage of control (TRV2), RNAi-1 (TRV-*PpePL1*), and RNAi-15 (TRV-*PpePL15*). Each values represents the means ± standard error of three replicates. Significant differences (*p* < 0.05) between means are indicated by different letters.

TA. XT plus texture analyzer was used to measure TPA parameters (adhesiveness, springiness, cohesiveness, gumminess, chewiness, and resilience). The adhesiveness of the control and RNAi fruit increased throughout the storage period, and the adhesiveness of the control fruit was higher than that of the RNAi-15 fruit except for 8 days (Figure 8). Except for the 8 days of storage, the cohesiveness, gumminess, chewiness, and resilience of RNAi-15 fruit during storage were higher than those of control fruit. In comparison, downregulated *PpePL1* in fruit had a smaller effect on fruit texture parameters than downregulated *PpePL15*.

### Downregulated *PpePL1* and *PpePL15* Affect Pectin Content and Cell Wall Structure

**Figure 9:**
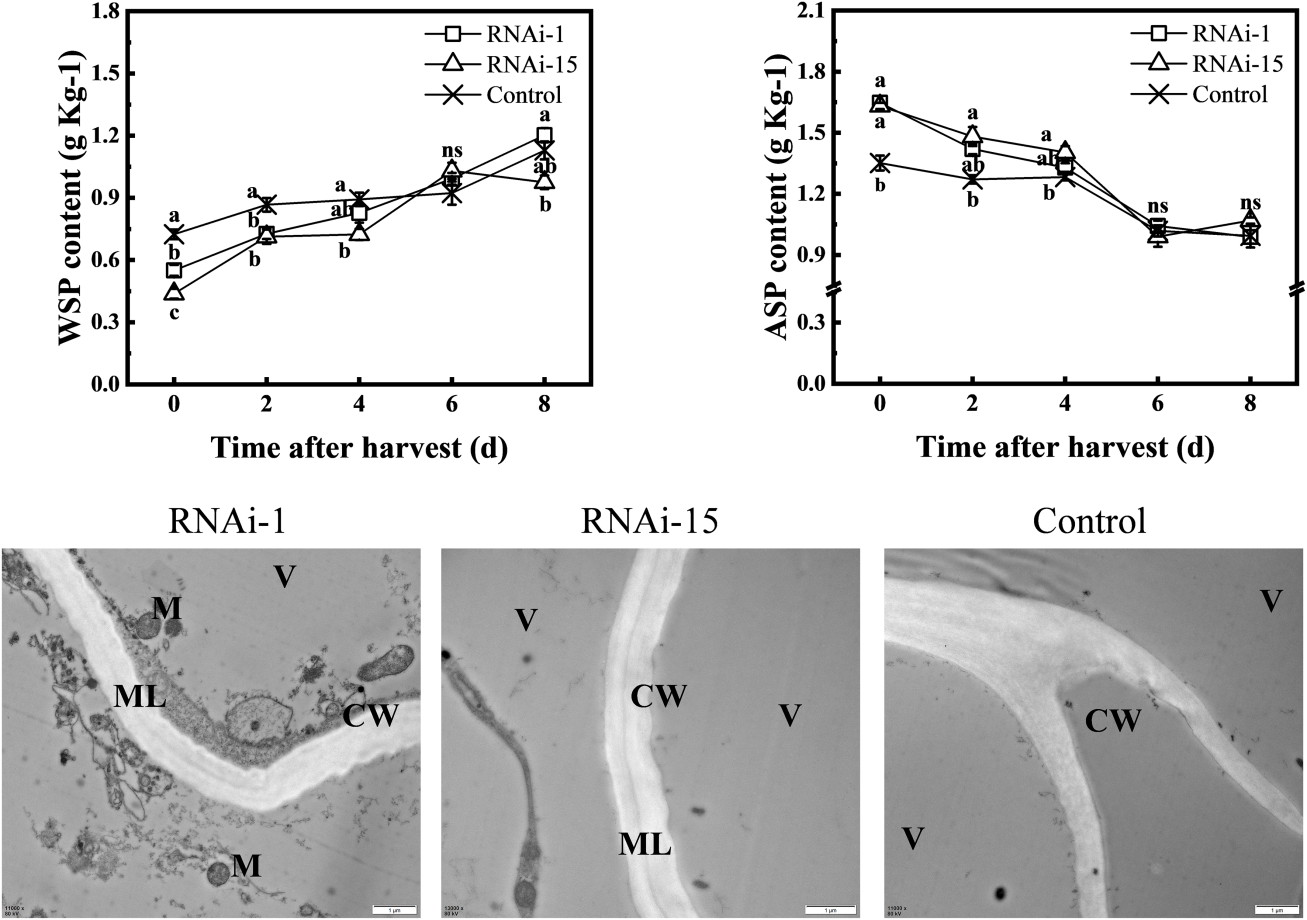
Change in contents of water-soluble pectin (WSP) and acid-soluble pectin (ASP), and cell wall ultrastructure. Cell wall ultrastructure was observed by transmission electron microscopy (TEM). The bars = 1 μM. ML, intercellular layer; CW, cell wall; M, mitochondrion; and V, vacuole. Each values represents the means ± standard error of three replicates. Significant differences (*p* < 0.05) between means are indicated by different letters.

In order to analyze the softening phenotype, the pectin content in RNAi and control fruit was studied in detail (Figure 9). At 0–4 days, the content of water-soluble pectin in RNAi-15 fruit was lower than that of the control, while the acid-soluble pectin content was obviously higher than that of control fruit. In RNAi-1 fruit, the WSP content was lower than that of the control at 0–2 days, and the ASP was higher than that of control fruit only at harvest day.

Furthermore, the results of transmission electron microscopy (TEM) showed that the dark gray middle lamella of the cell wall was clearly visible in RNAi fruit at 0 day, especially in RNAi-1 fruit (Figure 9). However, no middle lamella was observed in the cell wall of the control fruit. This demonstrated that pectin degradation was delayed after downregulated *PpePL1* and *PpePL15* expression.

## Discussion

The PL proteins are important for plant growth and development and have been isolated from a wide range of plant tissues, such as germinating seeds, developing flowers, ovaries, pollen, trichomes, and ripe fruit (Marin-Rodriguez et al., 2002; Singh et al., 2011; Jiang et al., 2014; Uluisik et al., 2016; Leng et al., 2017; Yang et al., 2020; Al Hinai et al., 2021; Seymour, 2021). To our best understanding, genome-wide analyses of the PL gene family have not been reported in peach. In this study, we identified the 20 *PpePL* genes and subsequently characterized the genes in terms of phylogenetic relationships, conserved motifs, and gene structure. And analysis of the expression level of *PpePL* gene members during the storage period in response to ethylene indicated that *PpPL1* and *PpePL15* genes may affect peach fruit ripening. Finally, VIGS was used to identify the functions of *PpPL1* and *PpePL15* in peach fruit softening. This study represents the comprehensive investigation of the peach PL gene family, providing the basic and potential values for further analyses in genetic improvement of fruit softening as well as fruit trees.

### Characterization of PL Genes in Peach

A total of 20 *PpePL* family members were identified in the peach genome. All of the PpePL proteins exhibited the conserved Pec_lyase_C domain with Ca^2+^ binding, catalysis, and substrate binding amino acid sites, which was similar to PL proteins in mango and maize (Turcich et al., 1993; Chourasia et al., 2006), indicating their conserved function in catalyzing pectin degradation. The number of peach PL family members is small, compared with 26 PL genes in *Arabidopsis* (Palusa et al., 2007), 30 in poplar (Bai et al., 2017), 46 in *Brassica rapa* (Jiang et al., 2013), 22 in tomato (Yang et al., 2017). It may be because the peach has not undergone whole genome duplication and alternative actions during evolution. Tandem duplication and segmental duplication events occur frequently in plants (Cannon et al., 2004; Moore and Purugganan, 2005). Some families, such as PG and DOF in peach, have undergone tandem duplication (Qian et al., 2016). Our results demonstrated that PpePL5, 6, 7, and 8 were identified as tandemly duplicated genes, which may be caused by the expansion of the PpePL gene family and contributed to their varied structures and functions.

The unrooted phylogenetic tree separated the PpePL genes into five different groups, consistent with a previous investigation in rose (Singh et al., 2011), tomato (Yang et al., 2017), poplar (Bai et al., 2017), and cotton (Sun et al., 2018). Except for group 5, the same phylogenetic group have similar exon–intron structures. In group 5, high diversity in the exon numbers of PpePL gene members was found, indicating their possible functional diversification (Palusa et al., 2007). The similarities and differences in the gene structures, domains, and motifs of PpePL might be related to conservation and subfunctionalization, as a result of their long evolutionary history and functional divergence in peach (Chen et al., 2017).

The majority of known PL genes involved in fruit softening were placed into groups 1 and 2. In group 1, PpePL1 was similar to FaPlC clusters, which are expressed only in fruit and mainly during the ripening stages of strawberries (Benitez-Burraco et al., 2003). PpePL20 had high homology with FaPLA and FaPLB, which are two ripening-related strawberry pectate lyase genes (Brummell et al., 2004). These results suggest that PpePL1 and PpePL20 may be associated with peach fruit ripening. In group 2, PpePL15 showed a closer relationship with SlPL, which is an important pectinase related to cell wall disassembly and fruit softening in tomato (Yang et al., 2017). Therefore, PpePL15 might also play an important role in regulating the peach softening process.

In addition, a number of known PL genes involved in the growth and development process were supplemented in the phylogenetic tree construction. It is suggested that PpePL9 belongs to group 3 and is in the same clade as StPLL, suggesting that PpePL9 may be responsible for cell expansion (Yang et al., 2020). In group 4, PpePL3, 4, 11, and 16 were similar to OsPLL3, which is involved in pollen development and male sterility (Zheng et al., 2018). In group 5, PpePL10 and PpePL18 may play the same role as ospse1 in premature senescence (Wu et al., 2013). It demonstrated that these PpePL genes may be involved in the growth and development process in peach.

### PL Family Members Related to Peach Fruit Softening

Fruit softening is involved in the disassembly of cell walls and the reduction in cell-to-cell adhesion (Wang et al., 2018). As one of the Cell wall-modifying enzymes, PL genes have been associated with the ripening of banana fruit as high levels of PL transcript accumulate predominantly in ripe fruit but not in unripe fruit (Pua et al., 2001; Marin-Rodriguez et al., 2003). Pectate lyase activity increased during fruit ripening in strawberry (Jimenez-Bermudez et al., 2002; Benitez-Burraco et al., 2003; Zhou et al., 2016), tomato (Uluisik et al., 2016; Yang et al., 2017), and peach (Ortiz and Lara, 2008). However, only a few PL genes were expressed in mature fruit. In banana and mango, it has been found that MaPel I, MaPel II, and MiPel1 is only expressed in fruit, but not in roots and leaves, suggesting that these genes play an important role in fruit ripening and softening (Marin-Rodriguez et al., 2003; Chourasia et al., 2006). In tomato, most of the PL genes have less expression even almost no transcript in the mature green fruit and breaker stage, same as peach (Yang et al., 2017). The PL gene expression is mainly confined to fruit ripening stages when fruit softening is occurring in banana (Marin-Rodriguez et al., 2003; Payasi and Sanwal, 2003). Increased transcript accumulation during ripening has also been reported for three PL genes in strawberry and the highest expression was observed in the red fruit stages (Jimenez-Bermudez et al., 2002; Benitez-Burraco et al., 2003). Similar to the results in banana, mango, and tomato, only five *PpePL* genes in peach fruit has transcript accumulate during ripening and softening including *PpePL1, 9, 10, 15, 18*. Among them, the expression level of *PpePL1*, *10, 15, 18* were upregulated during fruit softening in “ZFW” and “QJB” fruit, indicating that *PpePL1, 10, 15, 18* may be related to peach fruit softening.

Ethylene was the key hormone in controlling ripening in climacteric fruit, such as peach, which matured with dramatically increased respiration caused by an ethylene burst (Hayama et al., 2006, 2008). During the postharvest period, peach fruit treated with ethylene exhibited accelerated softening, and 1-MCP treatment delayed it (Qian et al., 2016). In our study, expression levels of *PpePL1* and *PpePL15* were drastically enhanced after ETH treatment and obviously downregulated after 1-MCP treatment. In addition, the expressions of *PpePL9, 10,* and *18* were also affected by the treatment of ethylene regulators to varying degrees. As in strawberry, the gene is under hormonal control and its induction is regulated by a rapid increase in ethylene production at the onset of ripening (Benitez-Burraco et al., 2003; Zhou et al., 2016). The same result was found in mango 1-MCP treated fruit where a delay in ripening resulted in a delay in transcript accumulation of *MiPel1* (Chourasia et al., 2006). In peach, PL activity of 1-MCP-treated fruit was inhibited, supporting the idea that PL plays an important role in peach fruit softening (Ortiz and Lara, 2008). Furthermore, *PpePL1* and *PpePL15* exhibited the highest transcription abundance during peach fruit ripening and softening, and had function in response to the ethylene signaling pathway, indicating that *PpePL1* and *PpePL15* may play a significant role in peach fruit softening.

### *PpePL1* and *PpePL15* Are the Key PL Family Members Contributing to Peach Fruit Softening

To further clarify the role of *PpePL1* and *PpePL15* in peach fruit softening, VIGS technology was utilized to downregulate the expression of *PpePL1* and *PpePL15* in fruit. Our results demonstrated that the expression peaks of *PpePL1* and *PpePL15* were both delayed, and PL enzyme activity was inhibited in RNAi (RNAi-1 and RNAi-15) fruit; and the firmness of RNAi fruit was higher than that of the control fruit at the early stage of storage; except at the end of storage, the cohesiveness, gumminess, chewiness and resilience of RNAi-15 were higher and adhesiveness was lower than those of control fruit. These experimental results indicated that fruit softening and texture change were delayed by downregulation of *PpePL1* and *PpePL15* expressions. The results are consistent with studies in strawberry, inhibiting the expression of pectate lyase genes resulted in a high increase in firmness of full ripe fruit and reduced the postharvest softening (Jimenez-Bermudez et al., 2002; Youssef et al., 2009). In tomato, downregulation of PL expression levels by antisense technology fruit resulted in prolonged fruit firmness (Uluisik et al., 2016). In this study, there was no significant difference in the firmness of RNAi fruit and control fruit in the last storage stage, suggesting that PL may not be the sole determinant of fruit softening. However, PL::RNAi fruit in tomato still maintain a relatively high firmness in all storage periods (Uluisik et al., 2016). The simultaneous downregulation of *FaplC* and *FaEG3* in strawberry showed similar fruit firmness to the lines that only inhibited *FaplC*, indicating that decreased softening of transgenic fruit was not correlated with the suppression of endo-b-1,4-glucanase gene expression (Youssef et al., 2012). These results demonstrated that although pectin lyase, as an enzyme that degrades the de-esterified pectin in the cell wall, is the main factor in fruit softening, there may be some differences in their functions in fruit with different softening characteristics.

Softening is a complex process associated with loosening of the cell wall, depolymerization of hemicelluloses, and disassembly of the pectin fraction (Brummell and Harpster, 2001). Peach fruit softening is closely related to the disassembly of cell wall components, especially pectin degradation in the middle lamella (Carpita and Gibeaut, 1993; Brummell et al., 2004). The softening of peach fruit was accompanied by an increase in WSP content during storage (Zhang et al., 2010). Our results showed that the downregulation of *PpePL1* and *PpePL15* expression in peach fruit caused a reduction in WSP content and delayed the reduction in ASP, indicating that reducing the conversion of ASP to WSP leads to a delay in the softening. TEM showed that RNAi fruit maintains a higher hardness than control fruit. These data suggest that softening of the RNAi fruit was delayed because of suppressed pectin solubilization and depolymerization. Taken together, it suggested that *PpePL1* and *PpePL15* were the core members of the PpePL gene family affecting peach softening. This is the same as results reported in tomato after the downregulation of the *SlPL* gene (Yang et al., 2017). In strawberry, pectate lyase silencing leads to decreased depolymerization of the strongly bound pectin fractions, alters the interactions between various components of the pectic matrix, and increases the solubility of a subset of pectin (Santiago-Domenech et al., 2008; Youssef et al., 2009).

## Conclusion

In this study, the pectate lyase gene family, consisting of 20 members in peach, was identified and characterized. Among them, *PpePL1* and *PpePL15* have function in response to ethylene signaling pathway with an abundant transcript accumulation, indicated that these likely to play a role in peach ripening. VIGS was used to clarify the roles of *PpePL1* and *PpePL15* during peach ripening. The results showed downregulated expression levels of *PpePL1* and *PpePL15* affected the fruit firmness and texture by depolymerizing pectin and degrading cell walls, meaning *PpePL1* and *PpePL15* are the core PL family members contributing to peach fruit softening.

## Data Availability Statement

The datasets presented in this study can be found in online repositories. The names of the repository/repositories and accession number(s) can be found in the article/**Supplementary Material**.

## Author Contributions

CZ conceived and designed the studies. ZX, JD, TK, and QL performed all plant physiological experiments. ZX, JD, and KL performed all molecular biology experiments. ZX and CZ wrote the manuscript. KS check the language. CZ, LX, JM, and DZ analyzed the results and modified the manuscript. All authors contributed to the article and approved the submitted version.

## Funding

This work was supported by grants from National Key R&D Program of China (No. 2019YFD1000203) and China Agriculture Research System (CARS-30-Z-16).

## Conflict of Interest

The authors declare that the research was conducted in the absence of any commercial or financial relationships that could be construed as a potential conflict of interest.

## Publisher’s Note

All claims expressed in this article are solely those of the authors and do not necessarily represent those of their affiliated organizations, or those of the publisher, the editors and the reviewers. Any product that may be evaluated in this article, or claim that may be made by its manufacturer, is not guaranteed or endorsed by the publisher.

## Footnotes

^1^ https://www.rosaceae.org/species/prunus_persica/genome_v2.0.a1

^2^ http://pfam.xfam.org/

^3^ https://www.ncbi.nlm.nih.gov/Structure/cdd/wrpsb.cgi

^4^ http://web.expasy.org/protparam/

^5^ https://solgenomics.net/

^6^ https://www.arabidopsis.org/

^7^ https://www.ncbi.nlm.nih.gov/

^8^ http://gsds.gao-lab.org/index.php
