# Supplementary material for: PpePL1 and PpePL15 Are the Core Members of the Pectate Lyase Gene Family Involved in Peach Fruit Ripening and Softening
Source: Front Plant Sci. 2022 Mar 25;13:844055. doi: 10.3389/fpls.2022.844055 (PMC8990770; doi:10.3389/fpls.2022.844055)
Supplement: Supplementary file 1 [file Data_Sheet_1.DOCX]

Table S1 The information of the primers used for RT-qPCR and cloning.

| Gene name | Primer （5’- 3’） | Purpose |
| --- | --- | --- |
| *PpePL*1 | F:GTGGTCGTGATGGTCGCTTCTATG | RT-qPCR |
|  | R:TCGCTTGAACACAATCCAGAGAGG |  |
| *PpePL*9 | F: GGAGATGTATGCTATCGGCGGTAG | RT-qPCR |
|  | R:CTGTGTTGGTGCCATCGGTGAT |  |
| *PpePL*10 | F: CCGCTCTATTTCGTCACCACTT | RT-qPCR |
|  | R:CATCATGTCCTCTACCGCCTTG |  |
| *PpePL*15 | F:TTGGGCATAGCGATTCTTATACCG | RT-qPCR |
|  | R:CCTACATCTTGGCATTCTCTGGAC |  |
| *PpePL*18 | F: AGCGGCATCCTCGTGTCAGA | RT-qPCR |
|  | R:ACACCCGTGGGCGTTAATCC |  |
| VIGS-*PpePL1* | F:CGGAATTCTGTCATCCAGAACGAGCCTCT | Cloning |
|  | R:GGGGTACCCCAGCAGCATCACCTCATTGT |  |
| VIGS-*PpePL15* | F:CGGAATTCCCAGCGTGATATGACAATCCAAC | Cloning |
|  | R:GGGGTACCGTGATTGAAGGCTATGGTGACTTG |  |
| c-*PpePL1* | F:ATGGCGGTGTTTGAGAGTAAGA | Cloning |
|  | R:CTAGCAAGGATGGCCTCTGC |  |
| c-*PpePL9* | F:ATGCAGAAGCGTAAAAACACAAC | Cloning |
|  | R:TAAGGGTAATGATAATAGAGCACCAC |  |
| c-*PpePL10* | F:ATGGCCTCGCTACCGTAC | Cloning |
|  | R:CTATACAGTTACCGCCTGCG |  |
| c-*PpePL15* | F:ATGGCGAGGCCCTCCTTA | Cloning |
|  | R:TCAGCAACGAGAACCCTTTCT |  |
| c-*PpePL18* | F:ATGGGCAACTCCCACGGC | Cloning |
|  | R:CTACGCGACTCCTGTCTGATCA |  |

Table S2 The FPKM values of PpePL genes in peach cultivar ‘QJB’ during storage.

|  | QJB 0d | QJB 2d | QJB 4d |
| --- | --- | --- | --- |
| *PpePL1* | 786.26 | 1172.25 | 1469.66 |
| *PpePL2* | 9.27 | 0.18 | 0.05 |
| *PpePL3* | 0.00 | 0.00 | 0.00 |
| *PpePL4* | 0.00 | 0.00 | 0.00 |
| *PpePL5* | 0.00 | 0.00 | 0.00 |
| *PpePL6* | 0.00 | 0.00 | 0.00 |
| *PpePL7* | 0.00 | 0.00 | 0.00 |
| *PpePL8* | 0.00 | 0.00 | 0.00 |
| *PpePL9* | 17.31 | 8.39 | 12.07 |
| *PpePL10* | 13.61 | 14.14 | 13.43 |
| *PpePL11* | 0.01 | 0.00 | 0.00 |
| *PpePL12* | 0.76 | 0.18 | 0.00 |
| *PpePL13* | 0.00 | 0.00 | 0.00 |
| *PpePL14* | 0.00 | 0.00 | 0.00 |
| *PpePL15* | 2684.66 | 4675.96 | 4464.71 |
| *PpePL16* | 0.00 | 0.02 | 0.00 |
| *PpePL17* | 0.00 | 0.00 | 0.00 |
| *PpePL18* | 44.32 | 45.24 | 81.14 |
| *PpePL19* | 5.31 | 0.51 | 0.06 |
| *PpePL20* | 0.17 | 0.00 | 0.00 |

Table S3 The sequence of protein used in phylogenetic trees.

| **Gene name** | **Gene ID** | **Protein sequence** |
| --- | --- | --- |
| SlPL1 | Solyc01g010740 | MAFISGNSILLVLFIWLASLVPHLHARIAEFDPYLEKQALEALNSSLEAYTNNPEEITNAFNKEVGNALLKYKSLRRHLKEKDKCMATNPIDRCWRCDKNWAENRMDLEECARGFGHKTTGGKNGKYYVVTDESDDNVQEPKPGTLRHAVIQEEPLWIIFEKSMVIKLRQELMITSDKTIDGRGVAVHIAYGAGLMIQFVHNVIIHNIRIQNIISTNGGMIRDSINHIGLRTVSDGDGISIFGSNHIWIDHCTLSECTDGLIDAIMASTAITISNCKFNYHNDVMLLGATDAFPQDAIMQVTVAFNRFGEGLIQRMPRCRWGFFHVVNNDYTHWQMYAIGGSAHPTIISQGNRFKASDNPNTKQVTKRDYATESEWKKWQWVSEGDSFLNGAYFVESGPQDKKKTALTKNHKIKFKPGSHAGRLTRFAGVLKCKPGIPC |
| SlPL2 | Solyc02g067450 | MTKSLFIIFFVLGAIIPAFNAHIGDFDEVWRRRAEEAIKFTHETYESEPANITLAFNQKTRHTVKELSTVVSKNETIRRALGTKKYDGPCTVTNPIDKCWRCDPNWADNRKKLVECSMGFGYKTTGGRDGEFYVVTDPSDDYTTPKPGTLRHAVIQKEPLWIIFEKNMKIKLHQELIMQGDKTIDGRGATVHITGGASIMIQYTKNVIIHGLHIHDIVEGSGGMVRDAVDHIGLRTKSDGDGAAVAADNSTRRHLAKKYKGPCMATNLIDKCWRCDPQWADNREKYADCAMGFGSKATGGKGGRVYVVSDNSDSDVENPAPGTLRHAVIQTEPLWIIFERHMHIKLQRELLMQGHKTIDGRGFNIHIEKGAGLKMQGVSNVIISNLHVHNIVITPGGMIRDSAEHVGIRSEDEGDGISLFSATDIWIDHVSMSRATDGLIDAVKGSTGITISNCHFTDHDKVMLFGANDNHVEDKKMQITLAYNHFGKRLDQRMPRVRFGFFHIVNNDYTHWMRYAIGGNNGATIISQGNRFIAQASPLIKEVTHREKVEESEWKNWTWLSIDDDMQNGAFFKTSGDQDALSKLQDLNLIPAEPSYKVGIITKFAGSLACTAGRPC |
| SlPL3 | Solyc02g080910 | MASLALVLALLFLCISTLQAEYYAPSKNYYAPSTTKKTMNVIDSCWRAKSNWAKNRYALADCAVGYGKAAIGGKNGAIYVVTNPSDDPVNPKPGTLRYGVIQSKPLWIIFNKDMVITLKNELMINSYKTIDGRGAKVEIAYGPCITIQRVSHVIIHGISIHDCKPGKRGIVRDSPVHAGHRNGADGDGIDIFQSTHVWIDHCYLARCTDGLIDVIHASTGVTISNNYFTQHDKVMLFGHNDNNKEDKIMKVTVAFNYFGPGLIERMPRVRLGYAHVANNRYEKWLMYAIGGSANPTIFSEGNYFLASKSTQVTKRESKNGWQNWKWRSSKDKFLNGAYFIPSGYGTTNPYYSKAQSFPVADGSMVPSLTADAGPLRCTSYKSSKSSDKDETKSLHDFFPSYDPQPHKNGLLNVIDSCWRWKGDWSSNRKALADCAIGFGSSTIGGKYGDIYIVNDSSDDPINPKPGTLRYGAIQSEPLWIIFKRDMVLTLENELMVNSYKTIDGRGAKVEISNGPCITLDYVTNVIIHGISIHDCKPGKKGMVRSSPEHVGERSGSDGDAISVFTSSNVWIDHCYLARATDGLLDVIHASTAVTISNNYFTEHDKVMLLGHNDEYTADRNMKVTVVYNHFGRELVQRMPRVRHGYAHVANNYYDQWLMYAIGGSADPTIFSEGNYFIAPDKAYNKEVTKRETEEKGWKSWKWRSSNDMFMNGAYFLPSGYGSIAPKYTRGQSFIVAHGAFTPSLTSNAGPLQCVVNEPC |
| SlPL4 | Solyc02g087670 | MGGTKLVYLSLLLFSTFLVINGHIGEFDEVWRRRAQEADEWAIKAYKPDPINVTLAFAKETGQALKEIKEAKLAVNGTRRELKGGGKKYDGPCSVTNPIDRCWRCQPDWADNRKRLADCAMGFAKGTTGGKAGEIYVVTDSSDDTSDPKPGTLRYGVIQKEPLWIIFAKSMTIRLHQELIVQSDKTIDGRGVNVHIANGAGFMLQYVKNVIIHGLRIHDIVVGSGGMIRDAMDHVGQRTQSDGDGISIFGSSNIWVDHVSMWSCYDGLVDAIEGSTAVTISNSHFTDHNEVMLFGASDSSSIDQRMQITVAYNHFGKRLIQRMPRCRWGFIHVVNNDYTHWNMYAIGGSQHPTIISQGNRFIAPPDMFKKEVTKRDYSPESVWKQWSWRSQGDLFMNGAFFVESGDPDWTQKHIQLFDGVVSASGDQVTWITRFSGALNCKPGEAC |
| SlPL5 | Solyc02g093580 | MSTLFFTFSLLLLAPLLVISSIQDPELVVQDVHRSINASLTRRNLGYLSCGSGNPIDDCWRCNPNWEKNRQRLADCAIGFGKNAIGGKNGRIYVVTDSGNDDPVNPKPGTLRHAVIQDEPLWIIFKRDMVIQLKQELVMNSYKTIDGRGASVHISGGPCITIHHTSNIIIHGINIHDCKQSGNGNIRDSPNHSGWWDVSDGDGISIFGGKNIWVDHCSLSNCHDGLIDAIHGSTAITISNNYFTHHDKVMLLGHSDSFTQDKGMQVTVAFNHFGEGLVQRMPRCRHGYFHVVNNDYTHWEMYAIGGSAAPTINSQGNRFLAPNEKYRKEVTKHEDAPESQWRSWNWRSEGDLMLNGAYFRQTGAGASSSSTYARASSLSARPSSLVGSITTNAGPVNCKKGSRC |
| SlPL6 | Solyc03g058890 | MGGPKIKYSFLFLCITFATIIPSLMAHIGHYDEVWRRRAEEAKEYARKIYEPHPENVTLAFNQKLRDTMKELKKVKGTHNNSTRRGLGTKKYTGPCMVTNPIDKCWRCDPNWADNRKKLADCAMGFGSKAIGGKDGEFYVVTDNSDDYNDPKPGTLRHAVIQKEPLWIIFKRGMNIRLHQEMIMQSDKTIDARGVNVHITKGAGITLQYIKNVIIHGLHIHDIVEGNGGMVRDAVDHIGIRTKSDGDGISIFGASNIWIDHVSMQRCYDGLIDAVEGSTGITISNGHFTDHNEVMLFGASDSSSIDQVMQITLAFNHFGKRLIQRMPRCRWGYIHVVNNDYTHWNMYAIGGSMHPTIITQGNRFIAPPDIFKKQVTKREYNPESVWMQWTWRSEGNLFMNGAYFTESGDPEWSSKHKDLYDGISAAPAEDVTWMTRFAGVLGCKPGKPC |
| SlPL7 | Solyc03g058910 | MEYSYRTKINVLFIVLILFVFAALGTAINAPRRKLTKKYRGPCMAVNSIDKCWRCDPFWAEDRQKMADCALGFGINAMGGKYGPYYIVTDNSDDDVVDPKPGTLRFGVIQKGPLWITFARSMRIRLTRELIVSSNKTIDGRGKYVHIANGAGIKIQSASNVIISNLRIHNIVPTAGGLLRESDDHLGLRGADEGDAISIFNSHDIWIDHISMSRATDGLIDAVAGSTNITISNCHFTDHEKVMLFGANDHAEEDRGMKITLAYNHFGKRLDQRMPRCRFGFFHLVNNDYTHWERYAIGGSSGATIISQGNRFIAEDKLLVKEVTYREKSTSSVEEWMKWTWITDGDDFENGATFTPSGDQNLLSKIDHLNLIQPEPSSKVGLLTKFSGALSCKIRRPC |
| SlPL8 | Solyc03g071570 | MSSQSLFYRAKMPPATPILLFIYLILSFFSPLIDSQLNLTLPNQHPYPESVVHQLHRRVNESRLLFQTIVRDSKCHSTGNPIDDCWQCDPNWAKDRQRLADCSIGFGQSAMGGKGGQIYVVRDSSDRDTVNPIPGTLRHAVVQEEPLWIVFAADMVIKLKHELIINNYKTIDGRGANVHITGNGCITLQYVSHVIIHNVHIYNCVPSGNTNIRSSPTHVGWRGKSDGDGISIFGSHNIWIDHCALSHCTDGLIDAIMGSTAITISNNYFSHHDDVMLLGHDDKYLPDSGMQVTIAFNHFGEGLVQRMPRIRRGYVHVVNNDFTHWQMYAIGGSANPTINSQGNRYTAPDDPNLKEVTKREDTDNGQWDEWNWRTDGDTMVNGAFFVPSGQGLSNQYTKAYSVDPKSALLINQLTANAGVLGGPRDNSISILPQVGGGTSEGSGRGHDQSNSGSVDLFGMIFSGTGASAAAPPTTTPILFSFFILLTLYIITITTQLSKPFLHLL |
| SlPL9 | Solyc03g113150 | MGLPKRNLFLFFVLVVLVVSIEAHIKEFDEVWKKRAQQAKKAARHAYNPNPKIVADHLNYQVDKAVRGSKSRRRDLQRYSGKCMATNPIDQCWRCDPNWARNRMKLTDCVLGFGRKTTGGKGGKIYVVMDNSDNELVNPKPGTLRHAVIQPEPLWIIFAKNMVIKLNQELIMTSNKTIDARGRQVHIAHGGGLMLQFIHNVIISNLHIHDTKAGAGGLIRDSVSHYGYRSKSDGDGISIFGSTNVWIDHISMSNCQDGLIDAVEGSTAITISNCHFTKHNDVMLFGASDTASGDSVMQITLAFNHFGHGLTQRMPRVRWGFVHVVNNDYTHWLMYAIGGSMHPTILSQGNRFIAPPNPNAKEVTKRDYAPENVWKNWVWKSQGDLMMNGAFFVESGDPKHAFLKGPDMITSKPGSSVSSLTQFSGSLKCIEGRPC |
| SlPL10 | Solyc04g010230 | MAALPYADVDSSLKALAGRAEGFGRFAIGGLNGPVYSVTTLADDGPGSLRDGCRKKEPLWIVFEVSGTIPLTSYLRVSSHKTIDGRGQRIILTGKGLQLKDCEHIIVCNLEFEGGRGHDVDGIQIKPNSRHIWIDRCSLRDYDDGLIDITRQSTDITISRCYFAQHDKTMLIGADPSHVGDRCIRVTIHHCFFDGTRQRQPRVRFGKVHLYNNYTRNWGIYAICASVESQIYSQCNIYEASQKKKAFEYYTEKAADKEEARSGLIRSEGDMFLNGAQGSLLTGIGGECVFHPSEFYPVWTLEPASDSLKGILHICTGWQSVSLPQEECARQLKPAR |
| SlPL11 | Solyc05g007080 | MASISFNNSVIFLFSLFCFSSIIPKLYANIADFDPYLEKRAEEALQSSLAAYNENPEGVTQIFNKEVGETLLNGTRRHLKEKDKEDKNDKDDKEGDEKSVDGCKAYNPIDKCWRCDKNWANNRKALADCARGFGHGTTGGKDGRFYVVTDPSDNNVEEPVPGTLRHAVIQEEPLWIIFEKSMIIRLKQELMINSNKTIDGRGVSVHVAYGGGLTLQFVHNVIVHNIRVHHILSKNGGMIRDSVKHIGLRTVSDGDAISLFGANRIWIDHCTLTKGADGLLDAIMASTAITISNCKFNHHNDVMLLGANDAFPQDKIMQVTVAFNRFGKGCIQRMPRCRWGFFHVVNNDYAKWEMYAIGGTANPTIISQGNRFKAADNPNTKEVTNRNGAPEALWRNWQWRSEGDLFKNGAFFRESGPEIKSTPFTEHTTIQFEPAKFVGRLTRKAGVIQCKIGKAC |
| SlPL12 | Solyc05g014000 | MGMPLSFLLLLTLLSPIFTFSSHVPDPEVIVQQVNEKINASRRNLGYLSCGTGNPIDDCWRCDPNWEKNRQRLADCAIGFGKQAIGGKDGKIYVVTDTSDDPVNPKPGTLRYGAIQDEPLWIIFSRDMVIKLKEELMLNSFKTIDGRGASVHIAGGPCITIQYVTNIIIHGLNIHDCKQGGNAYVRDSPQHYGWRTISDGDGVSIFGGSHVWVDHCSLSNCNDGLIDAIRGSTAITISNNYMTHHNKVMLLGHSDSFTRDKNMQVTIAFNHFGEGLVQRMPRCRHGYFHVVNNDYTHWEMYAIGGSASPTINSQGNRFLAPNDIFNKEVTKHEDAAESEWKNWNWRSEGDLMLNGAFFIRSGAGASSSYAKASSLSARPSTLVNSITMNAGALGCKKGKRC |
| SlPL13 | Solyc05g055510 | MISLTFILSFFFFLILSFSSLITSTFNNQTLSHQHPFPESVVQQVNRRINESISRRQISDTTVINYQCLTGNPIDDCWRCDPNWVDNRQQLADCAIGFGHGAVGGKGGRYYLVSDPSDYDTVNPTPGTLRHAVIQDEPLWITFAGDMIIRLKHELMINNYKTIDGRGANVHVTGGGCITLQYVTNVIIHNIHVYNCIPSGNSNIRQSTTQVGWRGMSDGDGISIYSSRNIWIDHCALSHCTDGLIDAIMGSTAITISNSYFTHHDKVMLLGHDDRYVPDVGMQVTIAFNHFGEGLVQRMPRCRRGYIHVVNNDFTEWQMYAIGGSANPTINSQGNRFTAPEDPNAKEVTKRVDVDERDWTEWNWRTEGDEMVNGAYFVPSGDGISNQYALASSMEPKSAFLIEQLTMNAGVIGVPRDTTVAMSFGGRTRTTIAANRSSSVRPSRSKDGDGGFLEKVFGSVASAGSSTSSPSSSTITILFSLLILYIITNNIGLL |
| SlPL14 | Solyc06g071020 | MVTLINRSSFFVILCTLFFSIEANIGEFDEVWRTRATQANKNAKESYNPNPEKVAANFNKHVHRSEEGSNSTRRDLHKYNGPCVATNPIDRCWRCDPHWAKNRQKLADCVLGFGHHTTGGKGGKIYEVTDPGDTDMVNPKQGTLRHAVIQPGPLWIIFAHHMVIKLNQELIMTGDKTIDARGQQVHITGGASLMLQYINNVIIHGLHIHDIKAGNGGLIRDSINHYGFRTKSDGDGISIYGSTNIWIDHVSMSNCDDGLIDAVQASTAITISNCHFTHHNDVMLFGASDSFKQDEILQITLAFNHFGQGLIQRMPRVRWGFVHAVNNDYTHWLMYAVGGSQQPTILSQGNRFIAPPNPNAKEVTKREYSPESVWKNWVWRSQGDLMMNGAFFVESGDPNHKFTTGPDMIHPRAGSDAGRLTRFSGSLNCIEGKPC |
| SlPL15 | Solyc06g071840 | MATSSISLIFLLSFLLLIPSLLASSPLQNHQYVVDQVDRSINVSRRNLGYLSCGTGNPIDDCWRCDPNWEKNRQRLANCAIGFGKNAIGGRDGKIYVVTNSGNDDPVNPKPGTLRYGVIQDEPLWIIFASDMVIQLKEELIMNSFKTIDGRGANVHIAGGPCITIQYVSNVIIHGIHIHDCKQGGNAMVRSTPQHYGWRTISDGDGVSIFGGSDIWIDHCSLSNCVDGLIDAIMGSTSITXSNNYMTNHDKVMLLGHSDSHVQDKNMQVTIAFNHFGEGLVQRIPRCRHGYFHVVNNDYTHWEMYAIGGSANPTINSQGNRFLASDIRFSKEVTKHENAPESEWKNWNWRSDGDLMLNGAFFVKSGAGASSNYAKASSLSAKSSSLISSLVSGAGALSCWKGSRC |
| SlPL16 | Solyc06g083580 | MAAALRLSCLCFLQLLIVIVLFINSSADNSIEQAEQFQSLSNSTMTNRLSGNDELRHEHAEDDPEAIASMVDMSIRNSTERRKLGYFSCRTGNPIDDCWRCDRNWQRNRRRLADCSIGFGRNAIGGRDGRFYVVTDSGDDDPVNPRPGTLRHAVIQDEPLWIVFKRDMHITLKQELIMNSFKTIDARGVNVHIANGACITIQFVTNIIIHGLHIHDCKPTGNALVRSSPSHYGWRTMADGDAISIFGSSHIWVDHNSLSNCADGLVDAIMGSTAITISNNYFTHHNEVILLGHSDTYVRDKVMQVTIAFNHFGEGLIQRMPRCRHGYFHVVNNDYTHWEMYAIGGSAAPTINSQGNRYLAPVNPFAKEVTKRVEPWEGSWRQWNWRSVGDLLLNGAYFTASGRAAPGSYARASSLAAKSSSLVGMITSNAGALS |
| SlPL17 | Solyc09g005850 | MGNHHGRFSHSKHHHHHHNQQGPPFFVPTQQTQNTQMGIALPYANVDSNLRGLAGQAEGFGGSSIGGRDGHVYQVLNLNDDGPGSLRDGCRKKEPLWIVFEVSGTIELRSHLSVSSFKTIDGRGQKIKLTGKGLRLKECEHVIICNLEFEGGRGHDVDGIQIKPKSRHIWIDRCSLSDYDDGLIDITKESTDITVSRCHFSKHDKTMLIGGDSSNCGDRCMRVTIHHCFFDGTRQRHPRVRFAKVHLYNNYTRNWGIYAVCASVESQIYSQCNIYEAGQKKVAFKYLTEKAADKEEACTGSIKSEDDLFVCGTQAGLLSTCSENNVFNPSEFYQTWTVERPSDDLKHYLQHCTGWQCIPRPN |
| SlPL18 | Solyc09g008380 | MFQISYIVVFLILTSFFPLGFGIFLNVTTLPGQHPDPESVALEVNRKVNASLSIFQSRRKMLSYTQSSCQTGNPIDDCWRCDHSWQLNRQRLADCAIGFGQYALGGKGGRYYVVTSSSDPDPVDPPPGTLRYGVIQEEPLWIVFSASMEIKLSEELIFNSHKTLDGRGVNVHITGGGCITLQYISNVIIHNIHVHHCYESGDTNVRSSPTHFGYRGKSDGDGISIFGSRDIWIDHCSLSNCKDGLIDVVMGSTGITISNNHFSHHNEVMLLGHNDDYLPDSGMQVTISFNHFGKKLIQRMPRCRRGYIHVVNNDFTRWEMYAIGGSGNPTINSQGNRYIAPFDPFAKEVTKRVDTDEGKWRNWNWRSEGDVMANGAYFVASGEEVEIKYEKAYSVEPKSADFIDQITLNAGVLIHRGSNSGKWTATTNNDTESAGDDGGGEDLVAISGDSDDDYGGDEESRSSTIYSNFSLLFNLLMALLALL |
| SlPL19 | Solyc09g061890 | MAIYWRSCLSLSALLILVLLVINVNPSHSAEEAEQFQSLKNLTMTNSLSEENVPNHEHAVDDPEKVVSMVDMSIKNSTERRKLGFFSCGTGNPIDDCWRCDRNWQRNRKRLADCAIGFGRNAIGGRDGKYYVVTDPNDDDPVNPRPGTLRHAVIQDRPLWIVFKRDMVITLKQELIMNSFKTIDGRGVNVHIANGACITVQFVTNIIIHGINIHDCKPTGNAMVRSSPSHFGWRTIADGDGISIFGSSHIWVDHNSLANCADGLIDAIMGSTAITISNNYFTHHNEVMLLGHSDSYVRDKIMQVTIAYNHFGEGLIQRMPRCRHGYFHVVNNDYTHWEMYAIGGSASPTINSQGNRYLAPANPFAKQVTHRVEQSDVWKHWNWRSEGDLMLNGAFFTPSGHGAAASYARASSLAAKASSLVGTLTSNAGALTCRRGYQC |
| SlPL20 | Solyc09g091430 | MAMASKKWLSFSFSFLLILLLLVGVYAAGVQQSNDGSDTRTVEKEQLLSSENSTMAVSLEDVEEKLSKHAVDDPEEVVAMVAQSIRNSTERRKLGYFSCGTGNPIDDCWRCDPNWQKNRKRLADCGIGFGRNAIGGRDGRYYVVTDNRDDDPVNPRPGTIRHAVIQEEPLWIVFSRDMVIQLKQELIMNSFKTIDARGYNVHIANGACLTIQFVTNIIIHGLHIHDCKPTGNAMVRSSTTHFGWRTMADGDAISIFGSSHVWVDHNSLSHCADGLVDAVMGSTAITISNNHFAHHNEVMLLGHSDSYTKDKQMQVTIAYNHFGEGLIQRMPRCRHGYFHVVNNDYTHWEMYAIGGSANPTINSQGNRYLAPANRFAKEVTKRVDTAAGQWKGWNWRSSGDLMLNGAYFTPSGAGASASYARASSLGAKSSSMVGAITSSAGPLACRRSRTC |
| SlPL21 | Solyc11g008140 | MFSLICILLFCLLVSFFPQIIASFNLTLPHQHPYPEVVVQEVQRKVNESISRRQLWNTTMNAITTQCETGNPIDDCWRCDPNWSKNRQRLADCAIGFGQNAIGGKNGKYYVVSDSSDLDTVNPTQGTLRHAVIQEEPLWIIFSGDMFIKLKHELIVNSYKTIDGRGAKVHITGNGCITLQYISNVIIHNIHIYNCLPSGNTDIRSTPTHVGHRGRSDGDGISIFSSRNIWIDHCALSHCTDGLIDAIMGSTGITISNSYFSHHDEVMLLGHDDSYSPDAGMQVTIAFNHFGVGLVQRMPRCRRGYIHVVNNDFTEWQMYAIGGSANPTINSQGNRYTAPVDANAKEVTKRVETDEGEWSGWNWRTDGDIMVNGAFFVPSGEGLSNQYAKASSVEPKSAAIIDQLTLNAGVFGATSESVDQIEDSVNSLKAKEYTLSISIFLVYFLFSSV |
| SlPL | Solyc03g111690 | MGTSSVFLLFLLSFLLLLPSLLASSNPQQVVDEVHRSINGSRRNLGYLSCGTGNPIDDCWRCDPNWEKNRQRLADCAIGFGKNAIGGRDGKIYVVTDSGDDNAVTPKPGTLRHAVIQTEPLWIIFARDMVIQLKEELIMNSFKTIDGRGASVHIAGGPCITIQYVTNIIIHGIHIHDCKQGGNAMVRSSPSHYGWRTVSDGDGVSIFGGSHVWVDHCSLSNCKDGLIDAIMGSTAITISNNYMTHHDKVMLLGHSDTYTQDKNMQVTIAFNHFGEGLVQRMPRCRHGYFHVVNNDYTHWEMYAIGGSADPTINSQGNRFLAPDIRFSKEVTKHEDAPESEWKNWNWRTDGDLMLNGAFFTRSGVRTGSSSYAKASSLSARPSSLVANLVSSSGALNCKKGSRC |
| AtPLL1 | AT3G09540 | MGNLHGIHRSHHGGSNFPGEAPNSPFPPPYTAAPAPAPAPSSPSDHHMTVGPYCHVDSSLRSLAGKAEGFGRAAVGGLNGPICHVTSLADEGPGSLREACKRPEPLWIVFDVSGTINLSSFVNVSSHTTVDGRGQKVKITGKGLRLKECENVIICNLEFEGGVGPDADAIQIKPKSHNIWIDRCSLKNYYDGLIDITRESTDITVSRCHFMNHNKTMLIGADTSHVTDRCIRVTIHHCFFDGTRQRHPRVRFAKVHLFNNYTRHWAIYAVGAGVESQIHSQCNIYEAGEKKTVFKYITEKAADKEKPGAGFVRSEGDLLLNGAKSCLSQGGERYVFSPIQHYSEWTVESPTDILKNYLKHSTGWQNLPLPLDRPPTTA |
| AtPLL2 | AT3G55140 | MTSLPYADADCSLRALAGRAEGFGRFAVGGLHGDLYVVTSLADDGPGTLREGGRRKEPLWIVFAVSGTINLNSYLSVSSYKTIDGRGQRIKLTGKGIRLKECEHIIICNLEFEGGRGHDVDGIQIKPKSRHIWIDRCSLRDYDDGLIDITRQSTDITVSRCYFAQHDKTMLIGADPSHVEDRCIRVTIHHCFFDGTRQRQPRLRFGKVHLYNNYTRNWGIYAVCASVEAQVFSQCNIYEAGVKKKTFEYYSEKAADKEEARTGLVRSENDLFLNGAQPSLMTGASEECVFHPSEHYPTWTVEPPSETLKQIMQICTGWQSLSRPSDHGVPK |
| AtPLL3 | AT5G09280 | MTGNIGKGVTQYKVTDPSDDPLNPKPGTLRYGATLVKGKKWITFKRNMKIKLHKPLLISSFTALDGRGASVHISGPACLIVYRATDVIIHGLKIHDCKAHPPSSVMGPDSKIIQLGHMDGDAIRLVTAKKVWIDHNTLYDCEDGLLDVTLGSTDVTVSNNWFRNQDKVMLLGHDDGYVKDKDMRVTVVFNHFGPNCNQRMPRVRHGYAHVANNYYQGWTQYAIGGSMSPRVKSESNYFVAPKSGSKEVLEKHGPITAQIKGSQSQTLNRLKYSLLQPERYTAPETPSVSTTTLNMTF |
| AtPLL4 | AT4G22080 | MTLFTVSCLLVVLFLCHSLVHAENNGYYGYTPTVANYLPEKPQNIMNPVDSCWRLKSDWAANRKDLADCVVGFGSSTLGGKKGNLYVVTNPYDNAQNPQPGSLRYGVIQAKPLWITFAKDMVITLENELMVNSYKTIDGRGAKVEIAYGPCITIQDVTNVIVHGISIHDCKPGKYGMVRSSPTHVGHRKGSDGDAIAIFGSSNIWIDHCYLASCTDGLIDVIHASTGITISNNYFTQHDKVMLLGHNDDFVQDVKMKVTVAFNHFGPGLVERMPRVRRGYAHVANNRYDKWIMYAIGGSADPTIFSEGNYFIASDKSNSKEVTKREVKGGWNNWRWRTSKDVFKNGAYFVPSGYGSISLPYSSAQRFTVAPGNLVPSLTADAGPLNCNRNGPCY |
| AtPLL5 | AT4G22090 | MTHFTVSCLLVALFLCQSLVHAAYNGYYGYSPAAAPYPAEEPQNIMNPVDSCWRLKSDWDVNREDLADCAVGFGSSTLGGKKGNIYVVTNPYDNAQNPHPGSLRYGVIQAKPLWITFAKDMVITLANELMVNSYKTIDGRGAKVEIAYGPCITIQDVTNVIVHGISIHDCKPGKSGKVRSSPTHVGHRKGSDGDAITIFGSSNVWIDHCYLASCTDGLIDVIHASTAITISNNYFTQHDKVMLLGHNDNFVKDVKMKVTVAFNHFGPGLVERMPRVRRGYAHVANNRYDKWIMYAIGGSADPTIFSEGNYFIASDKSYSKEVTKREVKGGWNNWRWRTSNDVFKNGAFFVPSGYGSIPLPYSSAQRFTVAPGNLVPSLTADAGPLNCNRNGPCY |
| AtPLL6 | AT1G11920 | MASLFLTIISLLFAAFSSSVVEAAYSNGYTIPKLLPNPIDSCWRRNPYWASNRRALADCAVGFGKSAVGGKYGSIYVVTNPSDDPENPRPGTLRYAVIQSKPLWITFARDMVIVLRNELIMNSYKTIDGRGAKVEIAYGPCITIQHVSHVIIHGISIHDCKPGKSGRVRSSPTHVGSRKGSDGDAIAIFDSSHIWIDHCFFSRCQDGLIDVLHASTAVTISNNYFTQHDKVMLLGHNDNNVEDKIMRVTIAFNHFGPGLIERMPRVRRGYAHVANNRYEKWQMYAIGGSADPTIFSEGNYFVASDDPSKKQVTKRIDSGYDWKRWKWRTSKDVFKNGAYFVPSGYGTVTPLYGRAERFPVSHGSLVPLLTSSAGPLHCYSGRIC |
| AtPLL7 | AT1G30350 | MASLVVIVSLLLAAFASPLLETAHSYNVTAPRVSLNPIDACWRRNPKWATNRQALAHCAVGYGKAAIGGKNGPIYVVTNPSDNPTRPSPGTLRYAVSQPKPLWITFARDMVIVLKSQLMINSYKTIDGRGAKVEIANGPCLRIRQVKHVIIHGISIHDCKADPNGMDGDGIRVFQSTHVWIDHCFLSRCHDGLIDVIVSSTAVTISNNYFTQHDKVMLLGHDDSYMGDKDMRVTIAFNTFGPGLIERMPRVRRGYAHVANNRYEKWQMYAIGGSANPIIFSEGNYFVAPEKRSSKQVTKRMMAGPDSKRWKWGTSRDVFMNGAFFGPPGVIVRPLYKGGEGFRVAHGSLVPSLTSSAGPLRCYVGRIC |
| AtPLL8 | AT1G14420 | MAAAFLNLGGYVFVFFSSFLAIVAPQVRGNVAVFDSYWTQRQSDALKQTIGSYDPHPLNVTNHFNYHVNIAVDASESRNDTRRELTQVRSGRKTHKSSGKCLAYNPIDNCWRCDRNWANNRKKLADCVLGFGRRTTGGKDGPIYVVKDASDNDLINPKPGTLRHAVTRDGPLWIIFARSMIIKLQQELMITSDKTIDGRGARVYIMEGAGLTLQFVNNVIIHNIYVKHIVPGNGGLIRDSEAHIGLRTKSDGDGISLFGATNIWIDHVSMTRCADGMIDAIDGSTAVTISNSHFTDHQEVMLFGARDEHVIDKKMQITVAFNHFGKRLEQRMPRCRYGTIHVVNNDYTHWEMYAIGGNMNPTIISQGNRFIAPPNEEAKQITKREYTPYGEWKSWNWQSEGDYFLNGAYFVQSGKANAWSSKPKTPLPNKFTIRPKPGTMVRKLTMDAGVLGCKLGEAC |
| AtPLL9 | AT2G02720 | MVNLGSYVFVFVALSLTVVVPSVQAHIAEYDEYWTQRQTNALRETLESYDPNPENVTDHFNYHAALAMETTGIVNETRRDLRQVGRGKKTTRRGGRFESLNAIDKCWRGDKNWDKNRKKLADCVLGFGRKTTGGKNGPIYVVTDPSDNDLLKPKPGTIRHAVTRDRPLWIIFARSMIIKLQQELIITNDKTIDGRGAKIYITGGAGLTLQFVRNVIIHNIHIKQIKRGAGGLIIDSEQHFGLRTVSDGDGINIFGATNVWIDHVSMTDCSDGMIDAIMGSTAITISNSHFTDHDEVMLFGGTNKDVIDKKMQITVAFNHFGKRLKQRMPRVRFGLVHVVNNDYTHWEMYAIGGNMNPTIISQGNRFIAPPIEDSKQVTKREYTPYPEWKSWNWQSEKDYFLNGAYFVQSGKANAWSATPKNPIPRKFAIRPQPGTKVRRLTKDAGTLGCKPGKSC |
| AtPLL10 | AT3G01270 | METARLFKLVCVICIASLIPTIRANVADETDEYWVNKANEARKHTLMAYHPDPYEIVDHFHERHYDNSTDVEGTEEEKAVASEEEDVIEMISSPTNSTRRSLTGRGKGKGKGKWSKLTGPCTASNPIDKCWRCQPDWARRRKKLVHCVRGFGYRTTGGKRGRIYVVTSPRDDDMVNPRPGTLRHAVIQKEPLWIVFKHDMSIRLSQELMITSDKTIDARGANVHIAYGAGITMQYVHNIIIHGLHVHHIVKSSGGLIRDSINHFGHRGEADGDGISIFGATNIWLDHISMSKCQDGLIDAIMGSTAITISNSHFTHHNDVMLLGAQNNNMDDKKMQVTVAYNHFGKGLVQRMPRVRWGFVHVVNNDYTHWELYAIGGSQGPTILSHGNRFIAPPHKQHYREVTKRDYASESEWKNWNWRSEKDVFMNNAYFRQSGNPHFKCSHSRQQMIKPKNGMAVSKLTKYAGALDCRVGKAC |
| AtPLL11 | AT5G15110 | MEMVRLSKLMFTFCIAVLIPTIRGNISELDEYWSQRADEAREFTLQAYHSDPYEIVDHFHERHYDNSTDVTTPEEDGDAKPEEEEKEFIEMLGSSTNSTRRSLRGKGKGKWSKLKGPCTASNPIDKCWRCRSDWAKRRKKLTRCVRGFGHRTTGGKRGRIYVVTSNLDEDMVNPKPGTLRHAVIQKEPLWIIFKNDMSIRLNQELLINSHKTIDARGANVHVAHGAGITMQFVKNVVIHGLHIHHISESSGGMIRDSVDHFGMRTRADGDGLSIYGSSNIWLDHISMSKCQDGLIDAIVGSTGITISNSHFTHHNDVMLLGAQNTNEADKHMQVTVAYNHFGKGLVQRMPRIRWGFVHVVNNDYTHWELYAIGGSQGPTILSHGNRFIAPPHKPHYREVTKRDYASEDEWKHWNWRSDKDVFMNGAYFRQSGNPQYKCAHTRQQMIKPKNGLAVSKLTKYAGALDCRVGRRC |
| AtPLL12 | AT5G04310 | MVAHERRIHNLQKPTCICIIWFCLLVSLSHHGRASSTSASIFNLSLPHQHPFPEHVVLNVQRKLNDSLSRRQLLTYQQDDGTTASSPIPSCITGNPIDDCWRCDPNWSANRQRLADCSIGFGQGTLGGKGGQFYLVTDSSDNDAANPIPGTLRHAVIQPEPLWIIFSSDMGIKLKHELIIGSYKTIDGRGTNIQITGHGCLTIQQVSHVIIHNVHIHHCKPSGNTLVASSPTHVGFRGVSDGDGISVSASHHIWVDHCSLGYCADGLIDVILASTAVTISNNYFSHHDEVMLLGHDDRYTADKGMQVTIAFNHFGEGLVQRMPRCRHGYIHVVNNDFTAWEMYAIGGSASPTINSQGNRYTAPIDPNAKEVTKRVDSNEKHWSGWNWRTEGDVMVNGAFFVPSGDGVSPAYARATSVQPKAAAIIDQLTVNAGVFGDPSGRNGQGGSFPGITNGGGTITRGYSKSGPAGGGSGSDSDDGLFTLIFGNNSGAVALRPGQVWSILLIIILYWYIPHHTRS |
| AtPMR6/AtPLL13 | AT3G54920 | MLLQNFSNTIFLLCLFFTLLSATKPLNLTLPHQHPSPDSVALHVIRSVNESLARRQLSSPSSSSSSSSSSSSSSCRTGNPIDDCWRCSDADWSTNRQRLADCSIGFGHGTLGGKNGKIYVVTDSSDNNPTNPTPGTLRYGVIQEEPLWIVFSSNMLIRLKQELIINSYKTLDGRGSAVHITGNGCLTLQYVQHIIIHNLHIYDCKPSAGFEKRGRSDGDGISIFGSQKIWVDHCSMSHCTDGLIDAVMGSTAITISNNYFTHHDEVMLLGHDDNYAPDTGMQVTIAFNHFGQGLVQRMPRCRRGYIHVVNNDFTEWKMYAIGGSGNPTINSQGNRYSAPSDPSAKEVTKRVDSKDDGEWSNWNWRTEGDLMENGAFFVASGEGMSSMYSKASSVDPKAASLVDQLTRNAGVFGGPRDDQGQSGNSYSPYGGDGGGGGSSGGSSGGGMDVMGGTTRGSSSSSGDDSNVFQMIFGSDAPSRPRLTLLFSLLMISVLSLSTLLL |
| AtPLL14 | AT5G55720 | MSIVCTFFLFLLNTSFAFAFAIPKPPIVRRLSTTVTSNSTASSCSANGNPIDECWRCDENWKDNRKNLADCAVGFGRDSIGGRAGEFYTVTDSGDDNPLNPTPGTLRYAATQDQPLWIIFDRDMVIQLKQDLQVASYKTIDGRGNNVQIAYGPCLTLYKVSNIIINNLYIHDCVPVKRNALSSLGGYSDGDGISIFESRDIWIDHCTLEKCYDGLIDAVNGSTDITISNSYMLNHNEVMLLGHSDEYSGDRDMRVTIAFNYFGEGLVQRMPRCRHGYFHIVNNIYRDWKMYAIGGSANPTIFSQGNVFIASNNQFTKEVTKRESADGDEEWKEWNWKSEGDEMVNGAFFTPSGKEDSPSYAKFSSMVARPASLLKTTHPSVGVLSCEIDQAC |
| AtPLL15 | AT5G63180 | MFRPNSLLIPSNLSTTKSQRNTMLNSSYLSFALIFFCCILFSALASSLPVSDPELVVEEVHRKINESISRRKLGFFSCGSGNPIDDCWRCDKDWEKNRKRLADCGIGFGKNAIGGRDGEIYVVTDPGNDDPVNPRPGTLRYAVIQDEPLWIIFKRDMTIQLKEELIMNSFKTLDGRGASVHISGGPCITIQYVTNIIIHGLHIHDCKQGGNTYVRDSPEHYGYRTVSDGDGVSIFGGSHVWVDHCSLSNCNDGLIDAIRGSTAITISNNYLTHHNKVMLLGHSDTYEQDKNMQVTIAFNHFGEGLVQRMPRCRHGYFHVVNNDYTHWEMYAIGGSANPTINSQGNRFLAPDDSSSKEVTKHEDAPEDEWRNWNWRSEGDLLLNGAFFTYSGAGPAKSSSYSKASSLAARPSSHVGEITIASGALSCKRGSHC |
| AtPLL16 | AT1G67750 | MRMTLVHLSLSLFSCLLLVLSPTFIASTPVSEPELVVQEVNEKINASRRNLGVLSCGTGNPIDDCWRCDPKWEKNRQRLADCAIGFGKHAIGGRDGKIYVVTDSSDKDVVNPKPGTLRHAVIQDEPLWIIFARDMVIKLKEELIMNSFKTIDGRGASVHIAGGACITVQYVTNIIIHGVNIHDCKRKGNAYVRDSPSHYGWRTASDGDAVSIFGGSHVWVDHCSLSNCADGLIDAIHGSTAITISNNYLSHHNKVMLLGHSDSYTRDKNMQVTIAFNHFGEGLVQRMPRCRHGYFHVVNNDYTHWQMYAIGGSAAPTINSQGNRFLAPNDHVFKEVTKYEDAPRSKWKKWNWRSEGDLFLNGAFFTPSGGGASSSYAKASSLSARPSSLVASVTSNAGALFCRKGSRC |
| AtPLL17 | AT3G53190 | MMLQRSCIVLFFSLFLLVPQMVFSMLNRTLLLIPHPDPELVAYQVQWKVNASITRRQALDTTDQAGSTPCITGNPIDDCWKCDPNWPNNRQGLADCGIGFGQYALGGKGGQFYFVTDSSDDDAVNPKPGTLRYGVIQEEPLWIVFPSNMMIKLKQELIFNSYKTLDGRGANVHIVGGGCITLQYVSNIIIHNIHIHHCYQSGNTNVRSSPTHYGFRTKSDGDGISIFGSKDIWIDHCSLSRCKDGLIDAVMGSTGITISNNFFSHHNEVMLLGHSDHYEPDSGMQVTIAFNHFGEKLIQRMPRCRRGYIHVVNNDFTQWEMYAIGGSGNPTINSQGNRYTAPTNPFAKEVTKRVETPDGDWKGWNWRSEGDILVNGAFFVASGEGAEMRYEKAYSVEPKSASFITQITFHSGVLGVGGRNNNLGMWTTTGSEGTSGLDSYNDYTDEMSGAGSTNRLSFSVLVFLLSSISYLVVFTSSTQMFML |
| AtPLL18 | AT3G27400 | MVSYSNNHFAYAFLLLLTIGNTLAFSSSLPDHVQDPNLVVDDVNRSVFNASRRSLAYLSCRTGNPIDDCWRCDPNWETNRQRLADCAIGFGKNAIGGRKGRIYVVTDPANDDPVNPRPGTLRYAVTQEEPLWIIFKRDMVIRLKKELIITSFKTIDGRGSSVHITDGPCLKIHYATNIIIHGINIHDCKPGSGGMIKDGPHHTGWWMQSDGDAVAIFGGKHVWIDHCSLSNCDDGLIDAIHGSTAITISNNHMTHHDKVMLLGHSDSYTQDKNMQVTIAFNHFGEGLVQRMPRCRHGYFHVVNNDYTHWEMYAIGGSASPTIYSQGNRFLAPNTRFNKEVTKHEDAPESKWRDWNWRSEGDMLLNGAYFRESGAEAPSTYARASSLSARPSSLVGSITTTAGTLSCRRGRRC |
| AtPLL19 | AT4G24780 | MKMQTKKLFITIVSFLLYAPLFLSSPVPDPESVVEEVHKSINASVAGRRKLGYLSCTTGNPIDDCWRCDPHWEQHRQRLADCAIGFGKNAIGGRDGRIYVVTDSGNDNPVSPKPGTLRHAVVQDEPLWIIFQRDMTIQLKEELIMNSFKTIDGRGASVHISGGPCITIQYVTNIIIHGIHIHDCKQGGNAMVRSSPRHFGWRTISDGDGVSIFGGSHVWVDHCSFSNCEDGLIDAIMGSTAITLSNNHMTHHDKVMLLGHSDTYSRDKNMQVTIAFNHFGEGLVQRMPRCRHGYFHVVNNDYTHWEMYAIGGSANPTINSQGNRFLAPNIRFSKEVTKHEDAPESEWKRWNWRSSGDLLLNGAFFTPSGGAASSSYAKASSLGAKPSSLVGPLTSTSGALNCRKGSRC |
| AtPLL20 | AT3G07010 | MAVTKLILFASALLLTALFIGVNASRSNETWHEHAVENPDEVAAMVDMSIRNSTERRRLGYFSCATGNPIDDCWRCDRKWQLRRKRLADCSIGFGRNAIGGRDGRFYVVTDPGDDDPVNPIPGTLRHAVIQDEPLWIIFKRDMVITLKQELIMNSFKTIDGRGVNVHIANGACLTIQYVTNIIVHGIHVHDCKPTGNAMVRSSPSHYGFRSMADGDAISIFGSSHIWIDHNSLSNCADGLVDAVMSSTAITVSNNFFTHHNEVMLLGHSDSYTRDKVMQVTIAYNHFGEGLIQRMPRCRHGYFHVVNNDYTHWEMYAIGGSAGPTINSQGNRFLAPVNPFAKEVTKREYTGESKWKHWNWRSEGDLFLNGAFFTRSGAGAGANYARASSLSAKSSSLVGTMTSYSGALNCRAGRRC |
| AtPLL21 | AT5G48900 | MAVTQILVVFASALLLSMFFTGVDSTRSNETWHEHAVENPEEVAAMVDMSIRNSTARRRLGYFSCSTGNPIDDCWRCDRRWQSRRKHLANCAIGFGRNAIGGRDGRYYVVSDPNDDNPVNPKPGTLRHAVIQEEPLWIVFKRDMVITLKEELIMNSFKTIDGRGVNVHIANGACITIQFVTNIIIHGIHIHDCRPTGNAMVRSSPSHYGWRTMADGDGISIFGSSHIWIDHNSLSNCADGLIDAVMASTAITISNNYFTHHNEVMLLGHSDTYTRDKVMQVTIAYNHFGEGLIQRMPRCRHGYFHVVNNDYTHWEMYAIGGSASPTINSQGNRYLAPRNRFAKEVTKRDYAGQWQWRHWNWRSEGDLFLNGAFFTRSGSGLGASYARASSLAAKSSSLVGVITYNAGALNCRGGRRC |
| AtPLL22 | AT3G24670 | MVIFSRSFLALSTTLIILALCINSSTMAQETEDLNSHSSSNSSTANKLPNDDGAWNEHAVKNPEEVAAMVDMKIKNSTERRRLGFFSCATGNPIDDCWRCDRNWHLRRKRLANCAIGFGRNAIGGRDGRYYVVTDPSDHDAVNPRPGTLRHAVIQDRPLWIVFKRDMVITLTQELIMNSFKTIDGRGVNVAIAGGACITIQYVTNIIIHGINVHDCRRTGNAMVRSSPSHYGWRTMADGDAISIFGSSHIWIDHNSLSNCADGLIDAIMGSTAITISNNYMTHHNEVMLMGHSDSYTRDKLMQVTIAYNHFGEGLIQRMPRCRHGYFHVVNNDYTHWVMYAIGGSANPTINSQGNRFLAPGNPFAKEVTKRVGSWQGEWKQWNWRSQGDLMLNGAYFTKSGAAAPASYARASSLGAKPASVVSMLTYSSGALKCRIGMRC |
| AtPLL23 | AT4G13210 | MVVARTLFSISATLIIFLALFLHVNAVQETREPKHESSRNTSTVDNLSDGEWHEHAVKDPEEIAAMVDMSIRNSTYRRKLGFFSSCSTGNPIDDCWRCDKKWHRRRKRLADCAIGFGRNAVGGRDGRYYIVTDPSDHDPVTPKPGTLRYAVIQDEPLWIVFKRDMVITLSQELIMNSFKTIDGRGVNVHIAGGACLTVQYVTNIIIHGINIHDCKRTGNAMVRSSESHYGWRTMADGDGISIFGSSHIWIDHNSLSSCADGLIDAIMGSTAITISNNYLTHHNEAILLGHTDSYTRDKMMQVTIAYNHFGEGLIQRMPRCRHGYFHVVNNDYTHWEMYAIGGSANPTINSQGNRFLAPGNRFAKEVTKRVGAGKGEWNNWNWRSQGDLMLNGAYFTSSGAGASANYARASSLAAKSSSLVGMLTSSSGALKCRIGTLC |
| AtPLL24 | AT3G24230 | MATSSLKLTSACFVLLFIFVGCVLTATNLRNNEISRSRKLKTEDSKSFNSSPMTTRLDGVVELNEHAVTDPDKVAHEVSNLIHMSEQNITARRKLGFFSCGNGNLIDDCWRCDRNWNKNRKHLADCGMGFGSKAFGGRNGSYYVVTDHSDDDVVNPKPGTLRHAVIQVEPLWIIFKRDMVIKLKQELIMNSFKTIDARGANVHIANGACITIQNITNVIVHGLHIHDCKRTGNVTVRSSPSQAGFRGTADGDAINIFGSSHIWIDHNSLSNCTDGLVDVVNGSTAITISNNHFTHHDEVMLLGHNDSYTRDKMMQVTVAYNHFGEGLIQRMPRCRHGYFHVVNNDYTHWKMYAIGGSANPTINSQGNRFAAPKNHSAKEVTKRLDTKGNEWMEWNWRSEKDLLVNGAFFTPSGEGASGDSQTLSLPAKPASMVDAITASAGALSCRRGKPCY |
| AtPLL25 | AT4G13710 | MASSSQKLISVCVAVLVVLALTAMIFRNSEISLSRKLKTEVIQSSNSSTMAAIRKLKTEEFQSLNSSTMAATRLDGEPQQQQHAVADDPDMVADEVAKLVQMSEQNRTARRKLGFFSCGTGNPIDDCWRCDRNWHKNRKRLADCGIGFGRNAIGGRDGRFYIVTDPTDEDVVNPKPGTLRHAVIQEEPLWIVFKRDMVIELKQELIMNSFKTIDARGSNVHIANGACITIQFITNVIIHGLHIHDCKPTGNAMVRSSPSHFGWRTMADGDAVSIFGSSHIWIDHNSLSHCADGLVDAVMGSTAITVSNNHFTHHNEVMLLGHSDSYTKDKLMQVTIAYNHFGEGLVQRMPRCRHGYFHVVNNDYTHWEMYAIGGSAEPTINSQGNRYAAPMDRFAKEVTKRVETDASEWKKWNWRSEGDLLLNGAFFRPSGAGASASYGRASSLAAKPSSMVDTITSTAGALGCRKGRPC |
| AtPLL26 | AT1G04680 | MAVLPTWLLAMMCLLFFVGAMENTTHDNISSLPRSDETEWNQHAVTNPDEVADEVLALTEMSVRNHTERRKLGYFTCGTGNPIDDCWRCDPNWHKNRKRLADCGIGFGRNAIGGRDGRFYVVTDPRDDNPVNPRPGTLRHAVIQDRPLWIVFKRDMVIQLKQELIVNSFKTIDGRGANVHIANGGCITIQFVTNVIVHGLHIHDCKPTGNAMVRSSETHFGWRTMADGDAISIFGSSHVWIDHNSLSHCADGLVDAVMGSTAITISNNHLTHHNEVMLLGHSDSYMRDKAMQVTIAYNHFGVGLIQRMPRCRHGYFHVVNNDYTHWEMYAIGGSANPTINSQGNRYAAPKNPFAKEVTKRVDTPASHWKGWNWRSEGDLLQNGAYFTSSGAAASGSYARASSLSAKSSSLVGHITSDAGALPCRRGRQCSS |
| PpePL1 | ppa005761m | MAVFESKRWVTGAFLAVLLVLCFVAAIAEISGNRNGGTEELQSSSNSSMAARVAEDDESFNKHAVDDPEEVVAMVDMSIRNSTERRKLGFFSCGTGNPIDDCWRCDSNWQKNRKRLADCGIGFGRNAIGGRDGRFYVVTDPGDDDPVNPRPGTLRHAVIQNEPLWIVFKRDMVIQLKQELIMNSFKTIDGRGVNVHIANGACITIQFVTNIIIHGLHIHDCKPTGNALVRSSPSHFGWRTMADGDAVSIFGSSHIWVDHNSLSNCADGLVDAVMGSTAITISNNHFTHHNEVMLLGHSDSYTRDKAMQVTIAYNHFGEGLIQRMPRCRHGYFHVVNNDYTHWEMYAIGGSAEPTINSQGNRYAAPTNPFAKEVTKRVETPTTQWKSWNWRSEGDLLLNGAYFTPSGAGASASYARASSLGAKSSAMVGAITSGSGALPCRRGHPC* |
| PpePL2 | ppa006665m | MAMPLSLLLLSLLLIPAFISSSPVQDPELVIQEVQKSINASRRNLGYLSCGTGNPMDDCWRCDPNWEKNRQRLADCAIGFGKHAIGGRDGKIYVVTDAGDHPVNPKPGTLRYGVIQDQPLWIVFKGDMVIKLKEELMMNSFKTIDGRGASVHIAGGPCITIQYVTNIIIHGLNIHDCKQGGNAYVRDSPSHYGWRTLSDGDGVSIFGGSHVWVDHCSLSNCRDGLIDAIHGSTSITISNNYMTHHNKVMLLGHSDSYTQDKNMQVTIAFNHFGEGLVQRMPRCRHGNFHVVNNDYTHWEMYAIGGSASPTINSQGNRFLAPNDRFNKEITKHEDAPQKEWSKWNWRSSGDLLLNGAFFTASGAGASSSYARASSLGARPSSLVSSLTAGAGSLKCRKGSRC* |
| PpePL3 | ppa019055m | MAKLFNSYILLFFFFLSFAAIIANAESDQPQQLDEYWKKRAEQAENYTMESYEPNPQRVTDEFNAEVGELIMGKNETRRNLKGQKKYVGPCKVTNPIDSCWRCDPNWANNRMKIVDCIQGFGRKTTGGKGGPIYVVTDPSDGDLVNPRPGTLRHAVIQKGPLWITFARNMVIKLQQELMVASDKTIDGRGANVNIFDGAGITIQFVKNVIITNLHIKQIKAKEGGIIRDSVDHFGQRTKSDGDGISIFGSSNIWIDHVSMENCTDGLIDAIMGSTGITISNCHFTKHNEVLLFGASNSYTQDKMMQITVAFNHFGKGLVQRMPRCRHGFFHVVNNDYTHWLMYAIGGSMNPTIISQGNRFIAGLNSATKEVTKREYTAEAEWKNWLWKSEGDLFMNGAFFVESGSPANLRADKLDMIPFKPGTYVTKMTKFAGALDCFVGKPC* |
| PpePL4 | ppa005744m | MATINKSYFFLFFFFLSFALVIASDTDGTDQEEVQLDIYWKQRAEEAKKEAMQSFETDPEQVTEEFNSNVGELLMKQNGTRRHLRGNKKYNGPCMATNPMDACWRCDKNWAKNRKRLADCVQGFGKKTTGGKAGPIYVVTDPSDLDLVNPKPGTLRHAVIQKGPLWIIFARNMVIRLQQELLVTSDKTIDGRGANVNIHNGAGITLQFVKNVIITNLHIKKIVPKQGGMIRDSVDHIGRRTKSDGDGISIFGSSNVWIDHVSMENCADGLIDAIMGSTAITISNSHFTHHNDVMLFGASNSYTQDKIMQITVAFNHFGQGLVQRMPRCRHGFFHVVNNDYTHWLMYAIGGSMNPTIISQGNRFIAPPNQGAKEVTKRDHTAEAEWKNWEWRSEGDLMMNGAVFVQSGSGKSKHPAKMDLMPFKPGTYVAKLTKFSGALDCFVGKPC* |
| PpePL5 | ppa016917m | MACHGCPNGNANIWCFVLALLITIASFAPNPSFAKKTKVDGLKLNVIDGCWRWNSDWRRNRQELALCSVGFSGKMSNNIGRDVIYYQVTEPSDSALDPKPGTLRYAVTMIKGKKWITFRRDMHIRLDKPLLISSFTAIDGRGASVHIAGNACLLVFQASNIIIHGLRIHHCRPQPPSSVMGPEGKIMPIGQVDGDAIRLVTASKVWIDHNTLYECQDGLLDVTRGSTHITISNNWFRDQDKVMLLGHDDGYFRDKNMRVTVVYNHFGPNCNQRMPRIRYGYAHVVNNLYREWSQYAIGGSMNPSVKSEANLFIAPKSGNKKEITWRKDSIGDKESWKFYSVGDIFENGASFVETGAGRAKPNYNREQTFPVVNAKSVRSLTRSSGALICIKRSRC* |
| PpePL6 | ppa021860m | MACHGCPNGNANIWRFVLCLLIAIAGFAPNPSLAKKTKVDGLKLNVIDGCWRWNSDWRSNRQELALCSVGFSGKMSNNIGRDVIYYQVTDPSDNALDPKPGTLRYGVTMIKGKKWITFQRDMRIRLDKPLLISSFTAIDGRGANVHIAGNACLLVFQASNIIIHGLRIHHCRPQPPSSVMGPEGKIMPIGQVDGDAIRLVTASKVWIDHNTLYECQDGLLDVTRGSTHITISNNWFRDQDKVMLLGHDDGYFRDKNMRVTVVYNHFGPNCNQRMPRIRYGYAHVVNNLYREWSQYAIGGSMNPSVKSEANLFIAPKSGNKKEITWRKDSIGDKESWKFYSVGDIFENGASFVETGAGRAKPNYNREQTFPVVNAKSVRSLTRSSGALICIKRSRC* |
| PpePL7 | ppa016255m | MACHGCPNGNANIWRFVLCLLIAIAGFAPNPSLAKKTKVDGLKLNVIDGCWRWNSDWRSNRQELALCSVGFSGKMSNNIGRDVIYYQVTDPSDNALDPKPGTLRYGVTMIKGKKWITFQRDMHIRLDKPLLISSFTAIDGRGANVHIAGNACLLVFQASNIIIHGLRIHHCRPQPPSSVMGPEGKIIPIGQVDGDAIRLVTASKVWIDHNTLYECQDGLLDVTRGSTHITISNNWFRDQDKVMLLGHDDGYFRDKNMRVTVVYNHFGPNCNQRMPRIRYGYAHVVNNLYREWSQYAIGGSMNPSVKSEANLFIAPKSGNKKEITWRKDSIGDKESWKFYSVGDIFENGASFVETGAGRAKPNYNREQTFPVVNAKSVRSLTRSSGALICIKRSRC* |
| PpePL8 | ppa016145m | MDCHGCPNGNANIWRFVLCLLIAIAGFAPNPSLAKKTKVDGLKLNVIDGCWRWNSDWRSNRQELALCSVGFSGKMSNNIGRDVIYYQVTDPSDNALDPKPGTLRYGVTMIKGKKWITFQRDMRIRLDKPLLISSFTAIDGRGANVHIAGNACLLVFQASNIIIHGLRIHHCRPQPPSSVMGPEGKIMPIGQVDGDAIRLVTASKVWIDHNTLYECQDGLLDVTRGSTHITISNNWFRDQDKVMLLGHDDGYFRDKNMRVTVVYNHFGPNCNQRMPRIRYGYAHVVNNLYREWSQYAIGGSMNPSVKSEANLFIAPKSGNKKEITWRKDSVGDKESWNTTTKKQKDDGKSPSCIWFFNHYKEKPFWRPPVLVAKNCQFVAKTL* |
| PpePL9 | ppa003600m | MEGRGLLASQVVVCCYYWAALERKISIKEESFMQKRKNTTAANNKQIPFGPPSLLFFLSSLLSRMLPTTCILLICLLSSLSPLTKASSLNLTLPHQHPNPEAVAQEVQRRVNASLSRRQMLSLELKEQQQCLIGNPIDDCWRCDSNWARNRQKLADCGIGFGQDAMGGKGGQIYIVTDSSDRDPANPVPGTLRHAVIQTEPLWIIFSADMTIKLKCELIVNSFKTIDGRGFNVHVTGGGCITLQYVSNIIIHNIHVHHCKPAGNTNVASSPTHVGWRGKSDGDGISLFGARKIWIDHCSLSYCADGLIDAIMGSTGITISNSYFAHHDEVMLLGHDDKYLPDSGMQVTIAFNHFGEALVQRMPRCRRGYIHVVNNDFTQWEMYAIGGSANPTINSQGNRYTAPQDQNAKEVTKRVDTNEGDWSDWNWRTDGDIMVNGAFFVPSGAGMSTQYARASSTEPKSVALIDRLTNNAGVFGDPRSSTSVSHPGDDGGGTITDGTNTGSEGSSGGDGDYFGMIFGNGAPPSSSSSSNTIFLSLLIIFILYVTINHGGALLSLPLLLTLL* |
| PpePL10 | ppa008409m | MASLPYADVDFSLRALSGRAEGFGRFAIGGLHGPLYFVTTLADDGPGSLREGCRKQEPLWIVFEVSGTIHLSSYLSVSSYKTIDGRGQRVKLTGKGLRLKECEHIIVCNLEFQGGRGHDVDGIQIKPNSRHIWIDRCSLRDYDDGLIDITRQSTDITISRCYFAQHDKTMLIGADPSHVGDRCIRVTIHHCFFDGTRQRQPRLRFGKVHLYNNYTRNWGIYAVCASVESQIYSQCNIYEAGNKKKTFEYYTEKAADREEAKSGFISSEGDLFLNGAQPCALTGFNQECMFHPSEFYPHWTMEAASDSLKTILQILTGWQSIHRPAEQAQAVTV* |
| PpePL11 | ppa005602m | MASKLDLVLFMISFCILVPSLRASIANDSNVGQFDAVWKERELKAEKAALKAYQPNPEKVTDDFNESVQEFDDLNFEDDDQVSNITGRHLLGKKYKGPCKATNPIDRCWRCRKDWANDRKKLANCVKGFGRRTRGGKRGGFYVVTDPSDSNVVDPKPGTLRHAVIQPQPLWIIFARSMTIRLTQELILTSHKTIDARGANVRIAHGAGITLQFVQNVIIHGLHIHDIVPGSGGTIRDSVDHFGTRTQSDGDGISIFGSSHIWLDHLSMWNCGDGLIDVIQGSTAITISNCHFTRHNDVLLFGASDSFAGDAIMQITVAFNHFGKGLVQRMPRCRWGFIHVVNNDYTHWLMYAIGGSSHPTIISQGNRFIAPPNHAAKEVTKRDYAPVDVWKNWQWRSEGDLMLNGAFFVESGVAKKNHGFGKLDMMTAKPGTFVTRLTRFAGTLGCRVGQKC* |
| PpePL12 | ppa006569m | MAGLSLLLIFLIITCFLTATFASSSQLHDPELVAQEVHRSINASRRNLGYLSCGTGNPIDDCWRCDPNWEQNRQRLADCAIGFGKDAIGGRNGRIYVVTDSGDDDPVNPRPGTLRHAVIQDEPLWIIFKRDMVVQLKQELVMNSFKTIDGRGASVHIAGGPCITIHYATNIIIHGINIHDCKQAGNGNIRDSPQHSGWWTISDGDGVSIFGGKHIWVDHCSLSNCHDGLIDAIHGSTAITISNNYMTHHDKVMLLGHSDSYTQDKGMQVTVAFNHFGEGLVQRMPRCRHGYFHVVNNDYTHWEMYAIGGSAAPTINSQGNRFLAPNTRFNKEVTKHEDAPESEWRGWNWRSEGDLMLNGAYFRQSGAGASSSYARASSLSARPSSLVGSITTTAGALICRKGSRC* |
| PpePL13 | ppa025559m | MASLSPLILLSCFLTYYIAPTSQAYSPGYYTTTPKTTMNVVDSCWRSKSNWAINRRALANCAVGFGQDAVGGKYGSTYVVTTSYDDPINPKPGSLRYGVIQTQPLWIVFAKDMVITLKNELIMNSFKTIDGRGAKVEIAYGPCITVQGVSHVIIHGISIHDCKPGKGGNVRSTPTHVGHRRGCDGDAISIFASSHVWVDHCFLARSSDGLIDVTHASTAVTITNNYFSQHDKVMLLGHNDNFSADKGMKVTIAFNRFGAGLIERMPRVRFGYAHVANNRYDEWKMYAVGGSANPTIFSEGNYFIAPETAYAKQVTKRESGGRWNNWKWRSSKDMFKNGAFFVQSGYGSCYPLYSKTQSFKVLDGSMVPALTSSAGPLRCFVGKAC* |
| PpePL14 | ppa016012m | MASLSRLILLSCFLTYYIAPTSQAYSPGYYTTTTPKTTINVVDLCLSSNSNWATNRRTLANWAVGFGQDVDDHVNPQPGSLRYGVIETQRLWIVFAKDMVITLKNKLIMNSFKTIDGRGAKVEIAYGPCITVGHRRGCDGDATSHVWVDHCFLARSSDGLIDVTHASTAVTITDNYFSQHDKVMLLGHNDNFSADEGMKVTIAFNRFGAGLIERMPRGFGYAHVANNRYDEWKMYAVGGSANPTIFSEGNYFIAPETAYAKQATKRESRGRWNNWICSKMVLFFVQSGYGSCYPLYSKTQSFKVLDGSMVPALTSSAGPLRCFVGKAC* |
| PpePL15 | ppa006392m | MARPSLGPSPLSLLSFLLFCLLTPTLIASRPLQQNPELVVQDVQRSINDSVSRRNLGYLSCGTGNPIDDCWRCDPNWEQNRQRLADCAIGFGKNAIGGRDGKIYVVTDSGDNDPVNPKPGTLRHAVIQDEPLWIIFQRDMTIQLKEELIMNSFKTIDGRGASVHIAGGPCITVQFVTNIIIHGLHIHDCKPGGNAMVRSSPEHYGWRTISDGDGVSIFGGSHVWVDHCSLSNCKDGLVDAIHGSTAITISNNYMTHHDKVMLLGHSDSYTEDKNMQVTIAFNHFGEGLVQRMPRCRHGYFHVVNNDYTHWEMYAIGGSANPTINSQGNRFAAPDIRFSKEVTKHEDAPESEWRNWNWRSEGDLMINGAFFTASGAGASSSYARASSLGAKPSSLVGSITTASGALSCRKGSRC* |
| PpePL16 | ppa005859m | MAMEAEKLNVVILCVFVIAITIPTTVRANIADFDEHWQQRAAEAKKAAHEAYHDDPIAVTEHFNKQVLDTFDYANNTRRNLNQKYKGPCMATNPIDRCWRCDPNWEKNRKRLADCALGFGRKATGGKLGPIYVVTDNTDADLVNPKPGTLRHAVIQNGPLWIIFARDMRIKLTEELLVASDKTIDARGANVHILDGAQISLQFVKNVIITNLHIHNNKPGNGGMIRDSINHFGQRTRSDGDGISMFGATNVWIDHVSLSNCADGLIDAIQGSTAITISNCHLTNHNDVMLFGSSDSNSQDQVMQITLAFNHFGKGLVQRMPRCRWGFFHVVNNDYTHWLMYAIGGSQHPTIISQGNRFIAPSNQATKEVTHRNNAQEGEWRSWNWRSENDLMMNGAFFVQSGSPIRNLPKADMIQAKPGSFVTRLTRFAGPLKCVKNKPC* |
| PpePL17 | ppa025917m | MGATIIRGKKWITFQRDMRIRLEKPLLISSFTAIDGRGPSVHIAGNACLVVFKASNIIIHGLRIHHCRSQAPSLVMGPDGKTMPLGQVDGDAIRLVTASKDGLLDVTRGSTDITVSNNWFRDQDKVMLLGHDDGYFRDKNMRVTVVYNHFGPNCNQRMPRIRYGYAHVVNNLYREWSQYAIGGGSMNPSVKSEANLFIAPKSRNNKEITWRKDSIGNKESWKFYSVGDIFENRASFIRATPPICS* |
| PpePL18 | ppa007270m | MGNSHGHRKRRNGPVSSKTDNSGFKYGPSDQSAQIPSSSAKKTSMVALPYAHVDASLRALAAQAEGFGRLAIGGLRGALYHVTTLADDGPGSLRDGSRKKEPLWIVFEISGTIHLSSYLNVSSYKTIDGRGQRIKLTGKGLRLKECEHVIVCNLEFEGGRGPDVDGIQIKPNSKHIWIDRCSLRDYDDGLIDITRGSTDITISRCHFSQHDKTMLIGSDPSHIDDRCIRVTIHHCFFDATRQRHPRVRFAKVHLYNNYTRNWGIYAVCASVESQIFSECNIYEAGQKKVAFKYLTEKAADKEVAMTGHIKSEGDLFITGTQAGLTPTGVEHSMFHPSQHYQTFTVGPPTDDLKHVLQHCTGWQSVPRPADQTGVA* |
| PpePL19 | ppa004881m | MVPRTCIVLLFLLCSFSSLATAFLNLTLPGQHPNPEEVVQEVHRKVNASLARRQMLQGTIKYQDSSCLTGNPIDDCWKCDPNWPNNRQSLADCAIGFGQYALGGKGGEYYIVTDSTDDDAVNPRPGTLRYAVIQTEPLWIVFPGNMLIKLSQELIFNSYKTLDGRGANVHIVGGGCITLQFISNVIIHNVHIHHCYPSGDANVRSSPTHYGYRTKSDGDGISIFGSKDIWIDHCSLSHCKDGLIDAVMGSTGITISNNYFSHHNEVMLLGHSDDYLPDTGMQVTIAFNHFGEELVQRMPRCRKGYIHVVNNDFTQWEMYAIGGSGNPTINSQGNRYTAPSNPNAKEVTKRVETAEGKWKDWNWRSEGDILVNGAFFVASGKGVEFNYEKAYSVEPKSAVLIDQLIMHAGALGVGGRDNNLGKWSSGSNGDGNGLGSGPDYTDDMSGSSSRNNPLPLSSTSTLFSFFISLSCLLFSYIIPDMHFTMRK* |
| PpePL20 | ppa006229m | MAVALSLFSLLSTMVLLQALFVSAENEKLVNSRSDEALNEHAVDNPDEIASMVDMSIRNSTERRNLGFFSCATGNPVDDCWRCDPHWQLHRKRLANCGIGFGRNAVGGRDGKYYVVNNPRDDDPINPRPGTLRHAVIQDQPLWIVFKRDMVITLKQELIMNSFKTIDGRGVNVHIAYGACITVQFITNVIIHGLHIHDCKPTGNAMVRSSPSHYGWRTIADGDGISIFGSSHIWIDHNSLSNCADGLIDAIMGSTAITISNNYFTHHNEVMLLGHSDSYTRDKLMQVTIAYNHFGEGLIQRMPRCRHGYFHVVNNDYTHWEMYAIGGSAEPTINSQGNRYLAPNNAFAKEVTHRVETNNWKHWNWRSEGDLLLNGAYFIASGAGAAGSYARASSLGAKSSSMVGTITAGAGVLNCRRGYQC* |
| MiPel1 | AAX88800 | MAVSPRWISSVCALLILCLFLGVKASTVKHELNYRLLNSKNTSIADSSDDSWSQHAVDNPEEVAAMVDISIRNSTERRRLGYFSCETGNPIDDCWRCDPKWHLHRKHLADCAIGFGRNAIGGRDGKFYVVSDSSDDNPVDPKPGTLRHAVIQDRPLWIVFKQDMAITLKQELIMNSFKTIDGRGVNVHIANGACITIQYITNVIIHGIHIHDCKPTGNAMVRSSPSHYGWRTMADGDGISIFGASHIWIDHNSLSNCADGLIDAIMASTAITISNNYFTHHNEVMLLGHSDSYTRDKQMQVTIAYNHFGEGLIQRMPRCRHGYFHVVNNDYTHWEMFAIGGSADPTINSQGNRYLAPSNPFAKEVTKRVDTSDGVWKSWNWRSEGDLLLNGAYFISSGARSAASYARASSLGAKSSSLVGALTSSAGAMSCRVGRQC |
| FaPlA | AAB71208 | MRLASSIATMHFYMTPLLLLLALLVCVSASVENGKPVQSRFVEVVEEPRSSFNSSMADRSNDHWNEHAVDNPEEIASLVDTSIRNSSTRRELGYFSCATGNPIDDCWRCDPQWQRHRKRPANCGIGFGRNAVGGRDGKYYVVSDPGHDDPVNPRPGTLRHAVIQDRPLWIVFKRDMVITLKQELIMNSFKTIDARGVNVHIAYGGCITIQFVTNVIIHGLHIHDCKPTGNAMVRSSPSHYGWRTMADGDGISIFGSSHIWVDHNSLSNCADGLIDAIMGSTAITISNNYFTHHNEVMLLGHSDSYTRDKQMQVTIAYNHFGEGLIQRMPRCRHGYFHVVNNDYTHWEMYAIGGSADPTINSQGNRYLAPNNRFAKEVTHRVQTTGRWRHWNWRSEGDLLLNGAYF |
| MaPel1 | AAF19195 | MAAFMFFLTIAAFTAPVYSSRAPLTSAAVRDPELVVQEVQRSLNVSRRRLGYLSCGTGNPIDDCWRCDPDWADNRQRLADCAIGFGKNAIGGRDGEIYVVTDSGDDDPVNPKTGTLRYAVIQEEPLWIIFKRDMVIQLKEELIMNSHKTIDGRGASVHISGGPCITIQYVTNIIIHGVHIHDCKQGGNAYVRDSPGHYGWRTVSDGDGVSIFGGSHVWVDHCTLSNCHDGLIDAIHGSTAITISNNYLSHHDKVMLLGHSDELTSDKSMQVTIAFNHFGEDLVQRMPRCRHGYFHVVNNDYTHWEMYAIGGSAAPTINSQGNRFLAPNDRFAKEVTKREDAQESEWKKWNWRSEGDQMLNGAFFTPSGAGASSSYAKASSLGARSSSLVGTITVSAGVLSCKKGSRC |
| MaPel2 | AAF19196 | MTAGLRWIPPLLLLLLGFLLVLNGSRGWIGSERSSGSRNGGASRRSLREASANATSADASLEERAVTRAAEAAVDDPEEVASTVLTTIINSTARRSLGYLSCGSGNPIDDCWRCDPDWHVNRKKLADCGIGFGRNAIGGRDGELYVVTDSGDDDPVNPRPGTLRYAVIQDVPLWITFKHDMEITLKEELIMNSFKTIDGRGVNVHIANGACITIQYITNVIIHGLHIHDCKPTGNAMVRSSPSHYGWRTMADGDAVSIFGSSHIWVDHCSLSNCADGLVDAVMGSTAITVSNNYFTHHNEVMLLGHTDSYARDSIMQVTIAFNHFGEGLIQRMPRCRHGYFHVVNNDYTHWEMYAIGGSANPTINSQGNRYLAPTNPFAKEVTKRVDTDQSTWKNWNWRSEGDLLLNGAFFTPSGAGASASYARASSFGAKPSSLVDTLTSDAGVLSCQVGTRC |
| MdPel | AAQ84042 | MPKMPRPSSGPSLLSPLLLLPLLSLLSPTLISSRPLHLQDPELVVQEVQRNISDSVSRRNLGYLSCGTGNPIDDCWRCDPNWEKNRQSLADCAIGFGKNAIGGRDGKIYVVTDSGDDDPVNPKPGTLRHAVIQDEPLWIIFQRDMTIQLKEELIMNSFKTIDGRGASVHIAGGPCITIQFVTNIIIHGLHIHDCKQGGNAMVRSSPRHFGWRTVSDGDGVSIFGGSHVWVDHCSLSNCKDGLVDAIYGSTAITISNNYMTHHDKVMLLGHSDSYTNDKNMQITIAFNHFGEGLVQRMPRCRHGYFHVVNNDYTHWEMYAIGGSADPTINSQGNRFAAPDIRSSKEVTKHEDAPESEWKNWNWRSEGDLMLNGAFFTASGAGASSSYARASSLGAKPSSLVGAITTASGALSCRKGSRC |
| ZmPl | AAA16476 | MAAVIRSRRRVSVFFYVVLAAAAAAAAAQASNNVTSDEEYWAERAEVARSRNLAAYVSDPVAATNRFNADVLRATTRRALARYDGPCMATNPIDRCWRCRADWATDRKRLAQCARGFGHRTVGGAAGKLYVVRDPSDDEMIIPRKGTLRHAVIQDRPLWIVFARDMVIELRQELIVNHNKTIDGRGAQVHIMFAQITLQNVQNVILHNLHIHDSKAHSGGMIRDSKRHYGLRTRSDGDGVSVLSSSNVWIDHVSMSSCSDGLIDVVNGSTAITVSNSHFTDHDHVMLFGASNDSPQDAVMQVTVAFNHFGRGLVQRMPRCRYGFFHVVNNDYTHWIMYAIGGNMNPTIISQGNRFIAPDDPNAKEVTKREYTPYKDYKEWVWKSQGDVMMNGAFFNESGGQNERKYDRFDFIPAKHGRYVGQLTRFAGPLKCIVGQPC |
| FaPlB | AAK66160 | MRSASSIATMHFYMTPLLLLLALLVCVSASVENGKPVQSRFVEVVEEPKSSFNSSMADRSNDDWNEHAVDNPEEIASLVDTSIRNSSARRELGYFSCATGNPIDDCWRCDPQWQRHRKRLANCGIGFGRNAVGGRDGKYYVVSDPGHDDPVNPRPGTLRHAVIQDRPLWIVFKRDMVITLKQELIMNSFKTIDARGVNVHIAYGGCITIQFVTNVIIHGLHIHDCKPTGNAMVRSSPSHYGWRTMADGDGISIFGSSHIWVDHNSLSNCADGLIDAIMGSTAITISNNYFTHHNEVMLLGHSDSYTRDKQMQVTIAYNHFGEGLIQRMPRCRHGYFHVVNNDYTHWEMYAIGGSADPTINSQGNRYLAPNNRFAKEVTHRVQTTGRWRHWNWRSEGDLLLNGAYF |
| VvPL | AAF63756 | MSLLFLFSLLPPILGSPAPVQDPEVVVQEVHRSINASRRNLGYFSCGTGNPIDDCWRCDANWDKNRQRLADCAIGFGKDAMGGKNGRIYVVTDSEDDDPVNPRPGTLRHAVIQDEPLWIIFKRDMVIKLKQELVMNSFKTIDGRGASVHIAGGPCITIHYASNIIIHGLHIHDCKQGGNANIRNSPHHSGWWTVSDGDGVSIFGGRHIWVDHCSLSNCHDGLIDAIHGSTAITISNNFMTHHDKVMLLGHSDSYTEDKNMQVTIAFNHFGEGLVQRMPRCRHGYFHVVNNDYTHWEMYAIGGSADPTINSQGNRFLAPNDRFKKAVTKHEDAPESEWRHWNWRSEGDLMLNGAFFLQSGAGASSSYARRSSLSARPSSLVGSITLGSGALGCRKGSRC |
| ZePL | CAA70735 | MATTILPLILFISSLAIASSSPSRTPHAIVNEVHKSINASRRNLGYLSCGTGNPIDDCWRCDPNWANNRQRLADCAIGFGKNAMGGRNGRIYVVTDPGNDDPVNPVPGTLRYAVIQDEPLWIIFKRDMVIQLRQELVMNSHKTIDGRGVNVHIGNGPCITIHYASNIIIHGIHIHDCKQAGNGNIRNSPHHSGWWTQSDGDGISIFASKDIWIDHNSLSNCHDGLIDAIHGSTAITISNNYMTHHDKVMLLGHSDSYTQDKNMQVTIAFNHFGEGLVQRMPRCRHGYFHVVNNDYTHWEMYAIGGSASPTIYSQGNRFLAPNTRFDKEVTKHENAPESEWKNWNWRSEGDLMLNGAYFRESGGRAASSFARASSLSGRPSTLVASMTRSAGALVCRKGSRC |
| FaPlC | AAK66161.1 | MSIRNSTERRKLGYFSCGTGNPIDDCWRCDPNWQKNRKRLADCGIGFGRNAIGGRDGRFYVVTDPNDDDPVNPRPGTLRHAVIQDEPLWIVFKRDMVIQLKQELIMNSFKTIDGRGVNVHIANGACITIQFVTNVIVHGLHIHDCKPTGNAMVRSSPSHFGWRTMADGDAISIFGSSHIWVDHNSLSNCADGLVDAVMGSTAITISNNHLTHHNEVMLLGHSDSYTRDKQMQVTIAYNHFGEGLIQRMPRCRHGYFHVVNNDYTHWEMYAIGGSADPTINSQGNRYAAPTNPFAKEVTKRVETSQTQWRGWNWRSEGDLLLNGAFFTPSGAGASAVYARASSLGAKSSAMVGTITASAGALGCRRGRTC |
| OsPLL3 | LOC_Os02g12300.1 | MDDQRLQWRKPGSFLLVAGVFLAAAAAVSNAGIGEFDEHWEKRRAAAEAAAEEVYKPDPFNVTNEFNHAVIRSTERGVLRRELSGKNSKYKGPCLATNPIDRCWRCRKDWATDRKRLARCAMGFGRGATGGVRGKIYVVTDPGDGDAANPRYGTLRWGAMQAAPLWITFAKSMVIRLTQELLVASDKTIDGRGAQVHIARGGAGITVQFARNVIITSLHVHDVKHSDGGAVRDSPTHIGPRTRADGDGISLFAATDVWVDHVSMSMCEDGLIDVVQGSTGVTISNSHFTNHNDVMLFGASDSYPQDKVMQITVAFNHFGRGLVQRMPRCRWGFFHVVNNDYTHWLMYAIGGGMSPTILSQGNRYIAPPNIAAKLITRHYAPEWEWKNWAWRSDGDLFMNGAYFQASNGAINRKVKGSDMVKPKPGSYVRRLTRFAGALSCRPGEPC* |
| OsPLL4 | LOC_Os04g05050.1 | MAEAAARRWAAALVLLVLLTGTVELIGGGGGSGGRRLLAGLRAGSTAAASGTRRWLRDSSWPATAAAAAAASRGDDGDGDEASSAAMTVPGAVDDPEEVVSQVHMSIRNSTARRKLGYLSCGTGNPIDDCWRCDPDWHKNRQRLADCGIGFGRNAIGGRDGKIYVVTDPSDDDAVNPKKGTLRYAVIRDEPLWIVFKRDMVITLKQELIMNSFKTIDGRGANVHIANGACITIQYVTNVIIHGLHIHDCRPTGNAMVRSSPSHYGWRTMADGDAVSIFGASHIWVDHCSLSNCADGLIDAIMGSTAITVSNNYFTHHNEVMLLGHSDSYVKDKAMQVTIAFNHFGEGLIQRMPRCRHGYFHVVNNDYTHWEMYAIGGSAEPTINSQGNRYLAPTNPFAKEVTKRVETAQTIWKGWNWRSEGDLLLNGAFFTPSGAGASASYSRASSLGAKSSSMVGTITSGAGALSCRGGSAC* |
| OsRBH1 | LOC_Os10g31910.1 | MAGPVESTRITLLLVAATLLLLPPPLAASLNSSLPDPAAVVADFHSKVATSRRRMQKADGGGGGGGGGCLTGNPIDHCWRCAGTDWRQDRQRLADCGIGFGRNAMGGKGGPVYVVTDPSDGDPVNPAPGTLRYGAIQEGPLWIVFAGDMTIRLNEELLVNSYKTIDGRGANVHVGAGGACITLQYVSNVIIHNIHVHDCVPAGNANVRASPTHYGWRTRSDGDGISLYSARDVWVDHCALSRCADGLIDAIMGSTAITVSNSYFSHHNEVMLLGHSDGYLPDSAMQVTIAFNHFGIQLVQRMPRCRRGYFHIVNNDYTAWEMYAIGGSASPTINSQGNRYIAPADPNAKEVTKRVDTEEGQWAGWNWRTEGDMMVNGAFFVPSGEGLEAIYDKASSTDPKSSALVDQLTAGAGVLGGPRDNGEAAAYAGVNYAGVGTGGGGGGGAGAGGMGYGYLGMVYGSGGNWSCRADLTLQLTSLFLALFALICLHPL* |
| LjNPL | AFD54063.1 | MAFPIILMFLLLTPTLIFSSPVQDPESVVQEVQKSIIEHRRKLGFYSCGTGNPIDDCWRCDPNWENNRKRLAECAIGFGRHAIGGRDGKFYLVTDPSDHAVNPKPGTLRHAAIQQEPLWIIFKHDMVIKLEMDLVMNSYKTIDGRGANVHIAGGPCIKVQRKTNIIIHGISIHDCKRGGSGYVSDSPNHQSWRGRSDGDGVTIYDSSNVWVDHCSLSNCFDGLIDVVHGSTAITISNNYMTHHNKVMLLGHSDSNKEDKKMQVTIAFNHFGEGLGGRMPRCRFGYFHVVNNDYTHWQKYAIGGSSSPTIFSQGNRFLAPNDEEHKEITKHFGSSENEWKNWNWRSEGDLMLNGAFFSPSGAGATSTYARASSMEARPPMLVASMTAGAGALRCKKDYMCY |
| OsDEL1 | LOC_Os10g31910.1 | MAGPVESTRITLLLVAATLLLLPPPLAASLNSSLPDPAAVVADFHSKVATSRRRMQKADGGGGGGGGGCLTGNPIDHCWRCAGTDWRQDRQRLADCGIGFGRNAMGGKGGPVYVVTDPSDGDPVNPAPGTLRYGAIQEGPLWIVFAGDMTIRLNEELLVNSYKTIDGRGANVHVGAGGACITLQYVSNVIIHNIHVHDCVPAGNANVRASPTHYGWRTRSDGDGISLYSARDVWVDHCALSRCADGLIDAIMGSTAITVSNSYFSHHNEVMLLGHSDGYLPDSAMQVTIAFNHFGIQLVQRMPRCRRGYFHIVNNDYTAWEMYAIGGSASPTINSQGNRYIAPADPNAKEVTKRVDTEEGQWAGWNWRTEGDMMVNGAFFVPSGEGLEAIYDKASSTDPKSSALVDQLTAGAGVLGGPRDNGEAAAYAGVNYAGVGTGGGGGGGAGAGGMGYGYLGMVYGSGGNWSCRADLTLQLTSLFLALFALICLHPL* |
| ospse1 | LOC_Os01g36620.1 | MGASCSSPFPPPPSPAESNAPSASAMHHPPHPAAGGGGPVMPYADADRTLRALAGAAEGFGRRAIGGLHGPLYRVTSLDDDGHGTLRQACRAHGPLWIVFDVSGDIHLRTYLRVTSHKTIDGRGQRVRLLGKGLQLKECRHVIVCNLQIEGGRGHDVDAIQIKPSSADIWIDRCSLADCDDGLVDITRGSTDVTVSRCRFSRHDKTMLVGADPSHTGDRGIRVTVHHCFFDGTRQRHPRVRFGRAHLYNNYTRGWGIYAVAAGVEAQVASQCNVYEAGAERKAVFRYVPERAADREEAEAGWVRSEGDAFLNGARPCLVDGGDAAVFRPEEYYERWTMEAASPALKEVVQLCAGWQPVPRPPGE* |
| RbPel1 | ADP09680.1 | MRLASSRATMRFYMTPLLLLLALLVSVAASIENDKPLQSRSVGLEEPKSSINSSMSTERSNDDWNEHAVDNPEEIASLVDTTIRNSTARRNLGFFSCATGNPIDDCWRCDPQWQRHRKRLANCGIGFGRNAVGGRDGRYYVVNDPGHDDPVNPRPGTLRHAVIQDRPLWIVFKRDMVITLKQELIMNSFKTIDARGVNVHIAYGGCITIQYVTNVIIHGLHIHDCKPTGNAMVRSSPSHYGWRTMADRDGISIFGASHIWVDHNSLSNCADGLIDAIMGSTAITISNNYFTHHNEVMLLGHSDSYTRDKQMQVTIAYNHFGEGLIQRMPRCRHGYFHVVNNDYTHWEMYAIGGSADPTINSQGNRYLAPTNRFAKEVTHRVETTGRWRHWNWRSEGDLLLNGAFFVQSGAGAAASYARASSLGAKSSSMIGSITAGAGVLNCRSGRQC |
| StPLL | PGSC0003DMT400060372 | MLSNTIILSFSFFLILSFSSLIIATFNNLTLPHQHPFPESIVQQVNRRINESISRRQIFDTTVINYQCLTGNPIDDCWRCDPNWVNNRQQLADCAIGFGHGAVGGKGGRYYLVSDPSDFDTVNPTPGTLRHAVIQEEPGNPIEDGGRCDPNWVNNRQQLADCAIGFGHGAVGGKGGRYYLVSDPSDFDTVNPTPGTLRHAVIQEEPLWITFAGDMIIRLKHELMINNYKTIDGRGANVHVTGGGCITLQYVTNVIIHNIHVYNCVPSGNSNIRQSTTQVGWRGMSDGDGISIYSSRNIWIDHCAFSHCTDGLIDAIMGSTAITISNSYFTHHDKVMLLGHDDRYVPDVGMQVTIAFNHFGEGLVQRMPRCRRGYIHVVNNDFTEWQMYAIGGSANPTINSQGNRFTAPTDPNAKEVILIYNRRV* |
| PtPL1-18 | Potri.015G087800.1 | MSLTPFPLFSQFLSSQRRQQTMANPSLCLLFLLSLLTPALISSSPVQDPELVVQEVHRAINASRRKLGYLSCGTGNPIDDCWRCDPNWEKNRQRLADCAIGFGKNAIGGRDGKIYVVTDSGNDDPVNPRPGTLRHAVIQEEPLWIIFARDMTIQLKEELIMNSFKTIDGRGASVHIAGGPCITIQYVTNIIIHGLHIHDCKQGGNAMVRDSPKHFGWRTVSDGDGVSIFGGTHVWVDHNSLSNCNDGLVDAIHGSSAITISNNYMTHHDKVMLLGHSDSYTQDKNMQVTIAFNHFGEGLVQRMPRCRHGYFHVVNNDYTHWEMYAIGGSANPTINSQGNRFVAPDIRFSKEVTKHEDAPESEWKHWNWRSEGDLLMNGAFFTASGAGASSSYARASSLGARPSSLVGTITVGAGALGCRKGARC |
| GhPel | ADB90478.1 | MARTMAMAIPSLPLLFLFTFLLLLPLLISSSPVQDPELVVQDVHRAINASRRNLGYLSCGTGNPIDDCWRCDPNWETNRQKLADCAIGFGKNAIGGRDGKIYVVTDSGDDDPVNPKPGTLRHAVIQDEPLWIIFARDMTIQLKEELIMNSFKTIDGRGASVHIAGGPCITVQYVTNIIIHGLNIHDCKQGGNAMVRDSPRHYGWRTISDGDGVSIFGGSHVWVDHNSLSNCKDGLIDAIHGSTAITISNNYMTHHDKVMLLGHSDSYTQDKNMQVTIAFNHFGEGLVQRMPRCRHGYFHVVNNDYTHWEMYAIGGSANPTINSQGNRFTAPDNRFSKEVTKHEDAPESEWKSWNWRSEGDLMVNGAFFISSGAGASSSYAKASSLGARPSSLVATITTNAGALNCKKGSRC |
| PtxtPL1-27 | ACB12931.1 | MTMAVPQKWVCVFSAVIVLLFVGVVATSRPDVVGISALTRNVEAEKEQSSSNSTMAARSQEEADALNEKAVAADPEEVVSMVEMNIRNSTERRRLGYFSCGTGNPIDDCWRCDPNWHKNRKRLADCGIGFGRNAIGGRDGRFYVVTDSSDHDPVNPRPGTLRHAVIQDAPLWIVFKRNMVIQLKQELIMNSFKTIDGRGVNVHIANGGCVTIQFVTNVIIHGLHIHDCKPTGNAMVRSSPSHYGWRTMADGDAISIFGSSHIWVDHNSLSNCADGLVDAVMGSTAITVSNNHFTHHNEVMLLGHSDSYTRDKQMQVTIAYNHFGEGLIQRMPRCRHGYFHVVNNDYTHWEMYAIGGSAEPTINSQGNRYNAPANPFAKEVTKRVDTAPGNWKNWNWRSEGDLLANGAYFTPSGAGASASYARASSLGAKSSSMVGAMTANSGVLGCRRGHQC |

Figure S1 Cloned sequences of 5 full-length coding sequences compared with computationally predicted sequences in peach genome. The red boxes indicate the differing bases.


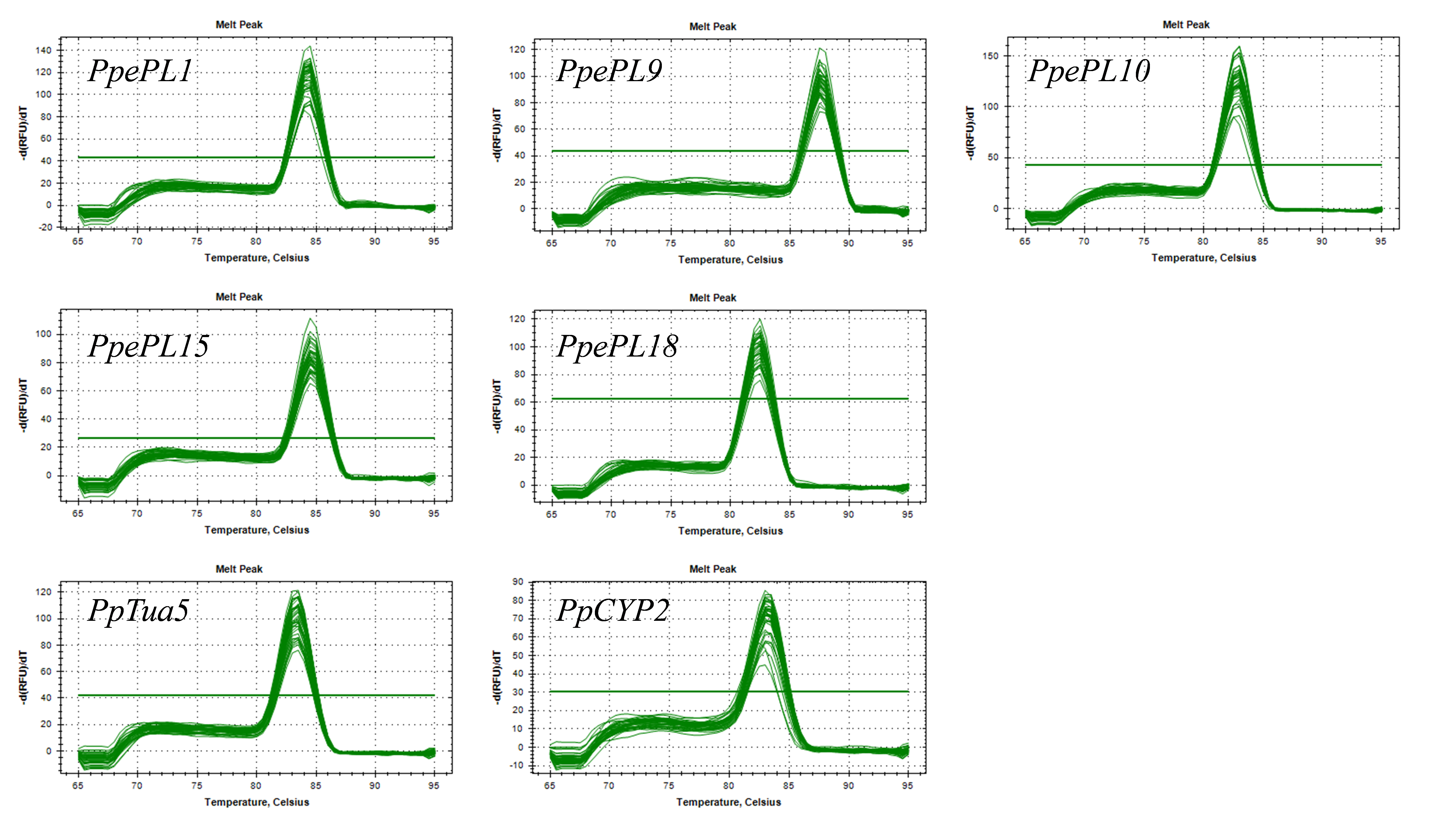


Figure S2 Melting curve of 5 *PpePL* genes and reference genes by RT-qPCR. A single peak indicated the specificity of primers.


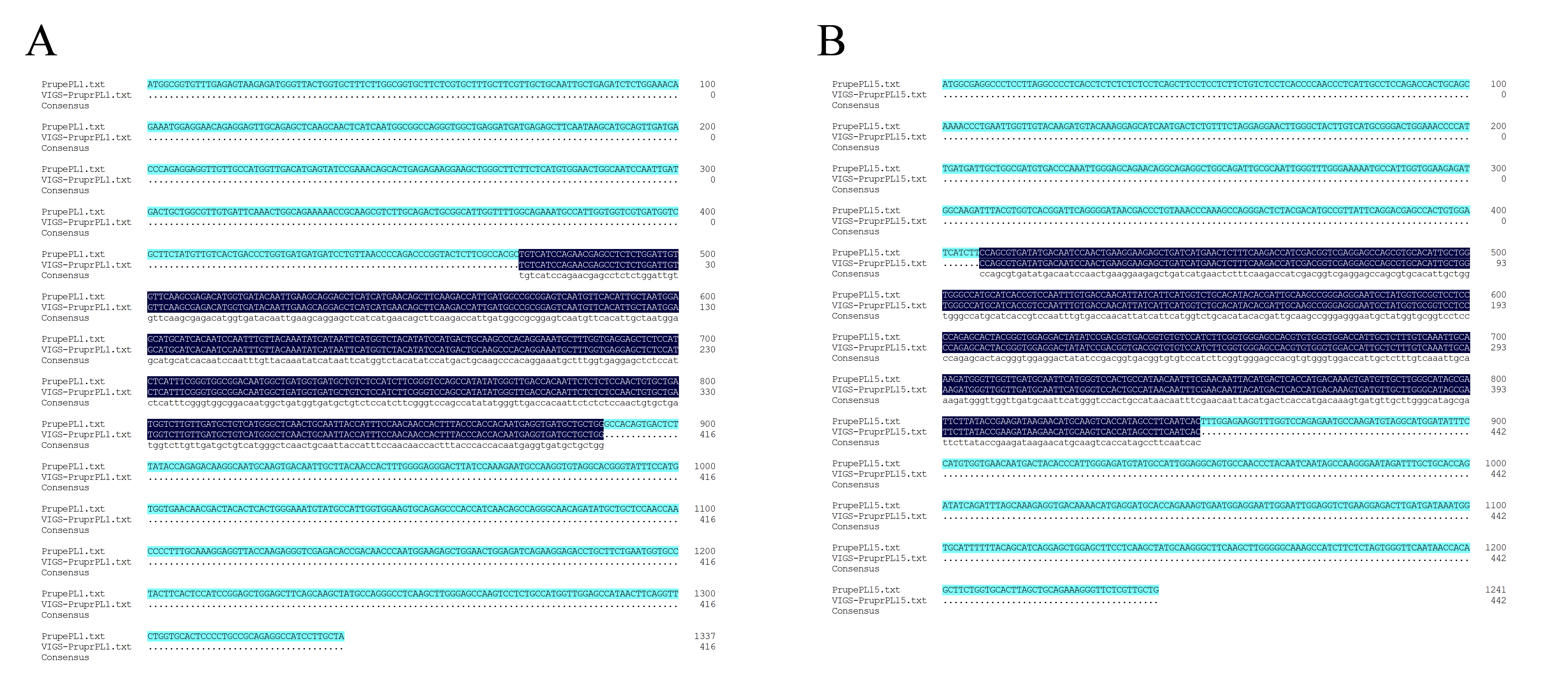


Figure S3 Vector construction of TRV-*PpePL1*(A) and TRV-*PpePL15*(B). Sequencing analysis of *PpePL1*and *PpePL15*.
